# Node-Solution Microenvironment Governs the Selectivity of Thioanisole Oxidation within Catalytic Zr-Based Metal–Organic Framework

**DOI:** 10.1021/acsami.5c09484

**Published:** 2025-07-18

**Authors:** Hafsa Abdul Ghuffar, Zaheer Masood, Bin Wang, Hyunho Noh

**Affiliations:** † Department of Chemistry and Biochemistry, The University of Oklahoma, Norman, Oklahoma 73019, United States; ‡ School of Sustainable Chemical, Biological and Materials Engineering, University of Oklahoma, Norman, Oklahoma 73019, United States

**Keywords:** metal−organic frameworks, oxygen atom transfer, microenvironment, thioether oxidation, Lewis acidic metal oxides

## Abstract

Lewis acidic metal oxides, including zirconia (ZrO_2_), are catalytically active toward oxidative reactions in the presence of sacrificial oxidants like *t*-butyl hydroperoxide (TBHP). The structural ambiguity and heterogeneity of the ZrO_2_ surface impose challenges to chemists in understanding the reaction mechanism down to atomic-level precision. The inorganic, Zr-oxo nodes of many crystalline metal–organic frameworks (MOFs) structurally mimic ZrO_2_. Herein, we report three novel findings: (A) Zr-based MOF, Zr-MOF-808 is catalytically competent in activating TBHP to induce oxygen atom transfer (OAT) reactions to a model substrate, thioanisole, at room temperature, (B) its reaction mechanism can be derived with greater structural precision owing to the crystallinity of the MOF, and (C) the node-binding agent and other reaction conditions significantly impact the selectivity between the singly oxidized methyl phenyl sulfoxide vs the doubly oxidized sulfone. These findings suggest that both the activity and selectivity of OAT reactions within Zr-MOF-808 are governed by the chemistry occurring at the interface of the node and the surrounding reaction medium. Implications of these findings in OAT reactions and other MOF/metal oxide-catalyzed relevant catalysis are discussed.

## Graphical abstract

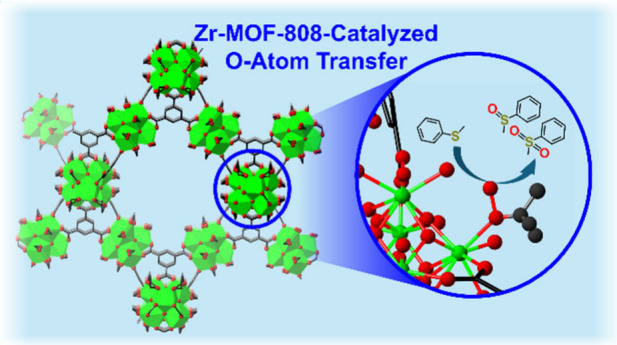

## Introduction

Oxygen-atom transfer (OAT) reactions are fundamental to many processes in biology, material corrosion, and industrially relevant chemical transformations.^1–4^ In heterogeneous catalysis, sacrificial oxidants first bind to the surface, and one or more of the O atoms are transferred to the substrate. Common sacrificial oxidants include O_2_ and more reactive peroxides, including but not limited to, hydrogen peroxide (H_2_O_2_) and *t*-butyl hydroperoxide (TBHP).^5–7^ Coordination of these oxidants to the catalyst weakens the bond(s) between the two O atoms and thus facilitates OAT to alkenes,^5,6,8^ phosphines,^7,9,10^ and thioethers^7,11,12^ to their corresponding oxidized products. The general OAT reaction between a substrate (X) and an oxidant (YO) is summarized below in eq 1.

1 X+YO⇌XO+Y

Activation of oxidants via O–O bond weakening requires the catalyst to withdraw electron density from the sacrificial oxidants. Hence, candidate catalysts often involve Lewis acidic metal oxides.^13^ Depending on the structure of the binding sites and their surrounding microenvironment, oxidants like TBHP can bind in η_1_-, η_2_-, or μ_2_-fashion, and thus present distinct reactivity (see Scheme 1).^14–18^ The surfaces of these metal oxides are often amorphous and can evolve over the reaction period. The structural evolution is more pronounced at elevated reaction temperatures and pressures.^19,20^ Thus, at any given time during the reaction, activated oxidants shown in Scheme 1 can coexist in various surface densities. It is this surface heterogeneity and ambiguity that have traditionally posed challenges to chemists in determining the structure–activity relationship in heterogeneous catalysis with atomic-level precision.

**Scheme 1:**
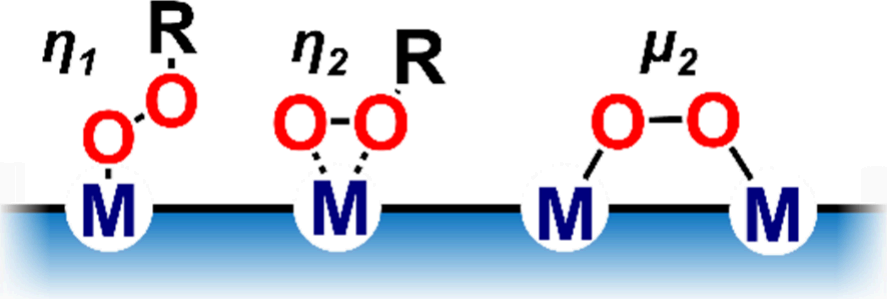
Schematic Illustration of Catalyst-Bound Oxidants

Metal–organic frameworks (MOFs) with Lewis acidic metal-oxo nodes within their porous structures are ideal for establishing atomically precise structure–activity relationships in OAT reactions.^21^ MOFs with hexanuclear Zr-oxo clusters are of particular interest, given their structural diversity where each inorganic node can be coordinated between four to 12 organic linkers.^22–24^ Depending on the node connectivity, each node can present terminal −OH/–OH_2_ groups that can be displaced by oxidants to induce OAT.^25^ Furthermore, Zr-based MOFs typically present higher chemical stability than other MOFs due to strong Zr-carboxylate interactions, and thus structural decomposition can be minimized.^26^ Due to these advantages, Zr-based MOFs and their composites have been employed in a wide range of applications, including but not limited to heterogeneous catalysis, energy storage, and others.^22,27–30^

Herein, we report our findings on the catalytic competency of a MOF, Zr-MOF-808,^31^ in thioanisole oxidation at room temperature using TBHP as the sacrificial oxidant (Schemes 2A and B). The observed reactivity was unexpected, as another Zr-based MOF with structurally similar nodes and in identical reaction conditions has been reported to exhibit little to no reactivity.^12^ This led us to determine the reaction mechanism and the catalytically relevant thermodynamics and kinetics. We further explored the role of reaction medium and other additives that dictate the microenvironment near the catalytic centers. These findings point toward the importance of the catalyst structure and surrounding microenvironment in dictating the activity and selectivity. Implications of these results will be benchmarked against the OAT reactivity of other Zr-based MOFs and metal oxides.

**Scheme 2:**
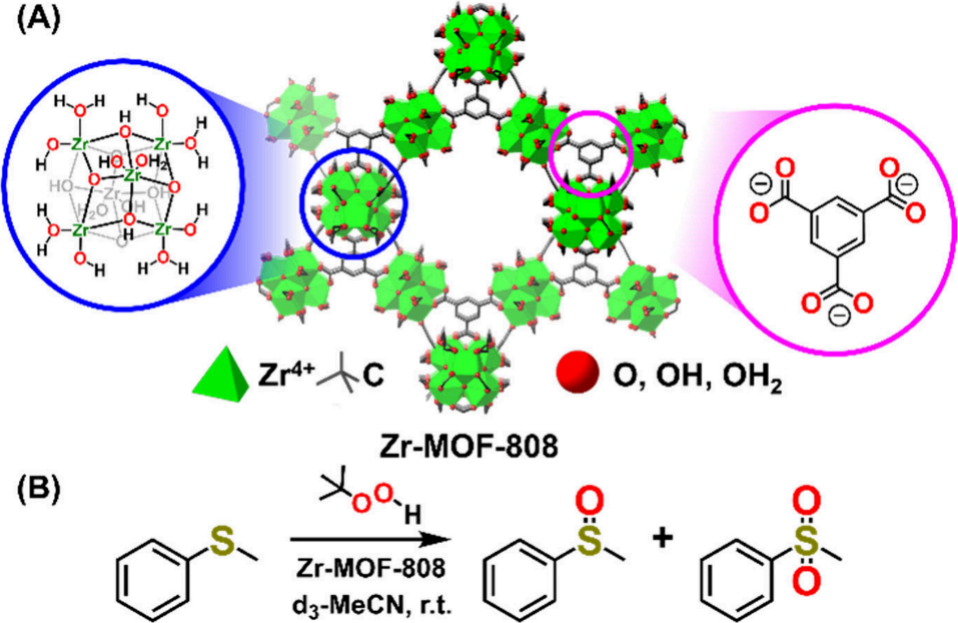
(A) Crystal Structure of Zr-MOF-808, and Its Inorganic Node and Organic Linkers; (B) Reaction Scheme of Zr-MOF-808-Catalyzed Thioanisole Oxidation

## Methods

Zr-MOF-808 was synthesized and acid-treated to remove formate units from the node according to the reported procedure.^32^ N_2_-adsorption–desorption isotherm reveals that the synthesized MOF has comparable porosity to that reported elsewhere, and the powder X-ray diffraction (PXRD) patterns coincide well with the expected pattern derived from the single-crystal X-ray diffraction analysis.^31^ Scanning electron microscopy (SEM) images show that, on average, the MOF-808 crystals were ∼400 nm in size; see Figures S1–3. ^1^H NMR of the MOF digested in *ca*. 1 M NaOD in D_2_O confirms that the MOF has a minimal amount of formate units within the lattice (Figure S4).

The catalytic oxidation of thioanisole followed the reported procedure.^12^ Briefly, a solution containing thioanisole (200 μL, 1.7 mmol), 1,1,2,2-tetrachloroethane as internal standard (360 μL, 3.4 mmol), *d*_3_-MeCN (1440 μL), and 10 mg of Zr-MOF-808 (equivalent to 7.6 μmol of Zr_6_ node) was added to a vial. In a separate vial, ∼3 M TBHP solution in *d*_3_-MeCN was prepared, assuming the concentration of TBHP in decane to be ∼5.5 M.

Into a standard NMR tube, 350 μL of the two solutions were added, and the ^1^H NMR spectrum of the resulting mixture was immediately measured; the resulting spectrum represented the kinetic profile at t = 0. The NMR tubes were constantly agitated during the reaction by attaching them to the arm of the conventional rotary evaporator. The reaction kinetics were monitored for at least 48 h. Figure S5 shows representative ^1^H NMR spectra at t = 0 and 48 h.

The concentrations of thioanisole, TBHP, and the amount of MOF were altered from those listed above to determine the apparent rate laws. Oxidation using the singly oxidized methyl phenyl sulfoxide was performed by preparing a substrate solution in *d*_3_-MeCN at identical concentrations.

Reaction in the presence of phenylphosphonic acid (PPA) followed the modified procedure of that reported previously.^33^ A solution containing PPA (87.4 mg, 0.55 mmol) in D_2_O was prepared, and 55.5, 165, or 330 μL of this solution was added to the reaction mixture to introduce one, three, or six equivalences of PPA with respect to the amount of active sites, respectively. The volume of *d*_3_-MeCN was adjusted to ensure that the total volume of the reaction mixture remains consistent as to above (assuming that the volume is additive).

All catalytic reactions were performed at least duplicate times, and 1σ of the two measurements are displayed as error bars here onward unless otherwise stated.

## Results

### Zr-MOF-808-Catalyzed Thioanisole Oxidation

We begin by describing Zr-MOF-808-catalyzed oxidation of thioanisole with ∼1.5 equivalence of TBHP in *d*_3_-MeCN, identical to that reported by Lee et al. previously.^12^ In the presence of ∼2.6 mol % of Zr-MOF-808, a measurable conversion of thioanisole to the corresponding oxidized products, methyl phenyl sulfoxide and methyl phenyl sulfone, was observed (Figure 1A). Here onward, these products will be simply referred to as sulfoxide and sulfone, respectively. For up to 10 h, the major product was sulfoxide, but its concentration decreased afterward as it was further oxidized to sulfone. The sulfoxide concentration was significantly reduced by 48 h; at this time point, the product selectivity is 24(4)% and 76(4)% for sulfoxide and sulfone, respectively (Table 1). As shown in Figure S6, in the absence of a MOF, the reaction does not proceed at the same rate, with only 5(2)% conversion of thioanisole solely to sulfoxide after 48 h. Thus, we attribute the observed thioanisole conversion primarily to the presence of Zr-MOF-808 in the reaction mixture.

**Figure 1:**
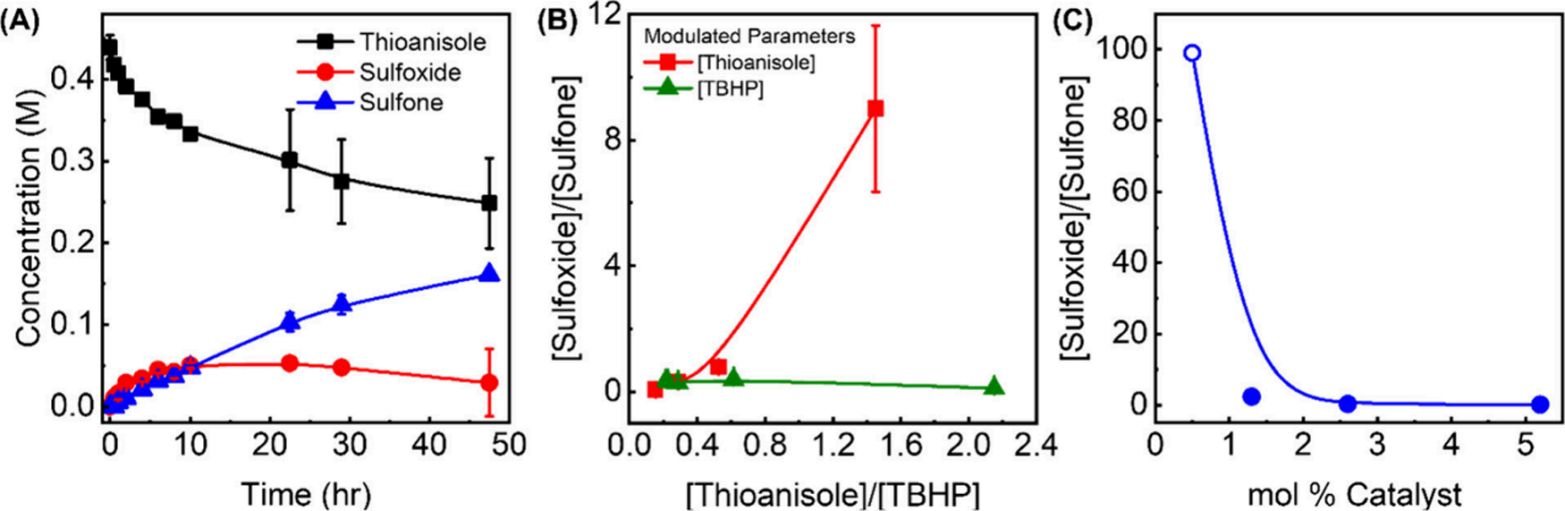
(A) Representative reaction profile of Zr-MOF-808-catalyzed thioanisole oxidation to corresponding methyl phenyl sulfoxide and sulfone (denoted sulfoxide and sulfone, respectively). Plots showing the molar ratio of the two products, [Sulfoxide]/[Sulfone], when (B) [Thioanisole], [TBHP], or (C) the amount of catalysts is modulated. The data points and error bars in (A–C) are the average of at least duplicate measurements. For (C) the data point at mol % of 0.5% assumes a 99:1 sulfoxide-to-sulfone ratio, as the detection of a small amount of sulfone was challenging.

**Table 1:** Table Showing Initial Rates of Thioanisole Conversion and Product Selectivity under Various Reaction Conditions

						Product Selectivity (%)	
Entry	[Thioanisole] (M)	Catalyst mol %^a^	[TBHP] (M)	Conversion (%)^b^	Initial Rate (mM hr^–1^)^c^	Sulfoxide	Sulfone
1	0.43	0	1.5	5(2)	–^d^	>99	<1^e^
2	0.43	2.6	1.5	47(1)	17(1)	24(4)	76(4)
3	2.18	2.6	1.5	18(5)	59(1)	90(10)	10(10)
4	0.79	2.6	1.5	21(1)	28(1)	44(5)	56(5)
5	0.23	2.6	1.5	69(3)	12(1)	6(1)	94(1)
6	0.43	2.6	2.2	64(1)	77(1)	24(1)	76(1)
7	0.43	2.6	1.9	57(1)	18(1)	22(1)	78(1)
8	0.43	2.6	0.7	33(3)	27(1)	28(3)	72(3)
9	0.43	2.6	0.2	23(1)	57(1)	10(1)	90(1)
10	0.43	5.2	1.5	68(1)	25(1)	9(1)	91(1)
11	0.43	1.3	1.5	24(3)	16(1)	70(10)	30(10)
12	0.43	0.5	1.5	12(3)	5.4(1)	>99	<1^e^

a All mol % calculations assume all Zr sites are catalytically addressable.

b These conversion values represent the amount of thioanisole converted after at least 48 h.

c Initial rates are calculated from the first derivative of the exponential fits within the first 30 min; see the SI for details.

d Initial rates when MOF is absent cannot be calculated as the reaction was too slow (see Figure S5).

e Under these conditions, sulfone was undetectable by ^1^H NMR. This does not rule out the presence of sulfone below the detection limit of the instrument.

To validate that Zr-MOF-808 is competent in the oxidation of sulfoxide to sulfone, sulfoxide was introduced as a *substrate* in otherwise identical reaction conditions. As shown in Figure S8, Zr-MOF-808 is indeed competent in conversion to sulfone; this reactivity is not observed in the absence of the MOF. Thus, we conclude that Zr-MOF-808 is serving as a heterogeneous catalyst in the thioanisole (and sulfoxide) oxidation reaction.

To further understand the reaction mechanism, we have conducted a series of reactions with varying concentrations/amounts of the substrate, the oxidant, and the MOF. Kinetic traces under these conditions, like that shown in Figure 1A, can be found in the SI (Figures S9–11). In this section, we will focus on thioanisole conversion and product selectivity. The next section will describe the correlation between reaction rates and mechanisms. An increase in thioanisole concentration led to a systematic *decrease* in selectivity toward sulfone – in other words, the catalyst prefers to yield sulfoxide over sulfone at higher thioanisole concentrations (Entries 2–5 in Table 1 and Figure 1B). This is expected as the molar ratio between thioanisole and TBHP also decreases, and thus, a smaller amount of oxidants is available for the oxidation of sulfoxide to sulfone. However, when the same molar ratio was decreased by changing the concentration of TBHP instead, the product selectivity toward sulfone remained high, between 70 to 90%; see Figure 1B and Entries 2, 6–9 in Table 1. As described later, these seemingly contradictory trends indicate thermodynamically favorable binding and activation of TBHP at the nodes of Zr-MOF-808. Product selectivity is also sensitive to the amounts of MOFs available in the reaction mixture (Entries 2, 10–12 in Table 1 and Figure 1C). This further corroborates that Zr-MOF-808 is indeed necessary to catalyze the observed reaction.

### Apparent Rate Laws

In this section, we describe how the initial rates of thioanisole conversion depended on the amounts of substrate, oxidant, and catalyst. All kinetic traces of thioanisole conversion were modeled using a *single* exponential function, as reported previously.^34^ This method enabled calculations of initial rates that are likely to have reduced diffusive complications; see Figure S13 and the relevant section in the SI for more details. The determined rates can be found in Table 1. An increase in thioanisole concentration led to an increase in reaction rate. The apparent reaction order with respect to thioanisole concentration was observed to be 0.7 (Figure 2A). This noninteger reaction order can be ascribed to diffusive complications (even though initial rates were used to minimize this effect).^35–37^ Furthermore, in all concentrations, quantitative conversion of thioanisole was not observed. Together with the noninteger reaction order, these may indicate inhibition of catalysis by products or others, and/or decomposition of Zr-MOF-808. The PXRD pattern of Zr-MOF-808 after catalysis indicates the retention of porosity and crystallinity (see Figure S2). SEM images of Zr-MOF-808 after catalysis also confirmed no change in crystal morphology (Figure S3). Thus, we believe this decrease in rate must be due to other reasons. We describe these in detail in the Discussion section.

**Figure 2:**
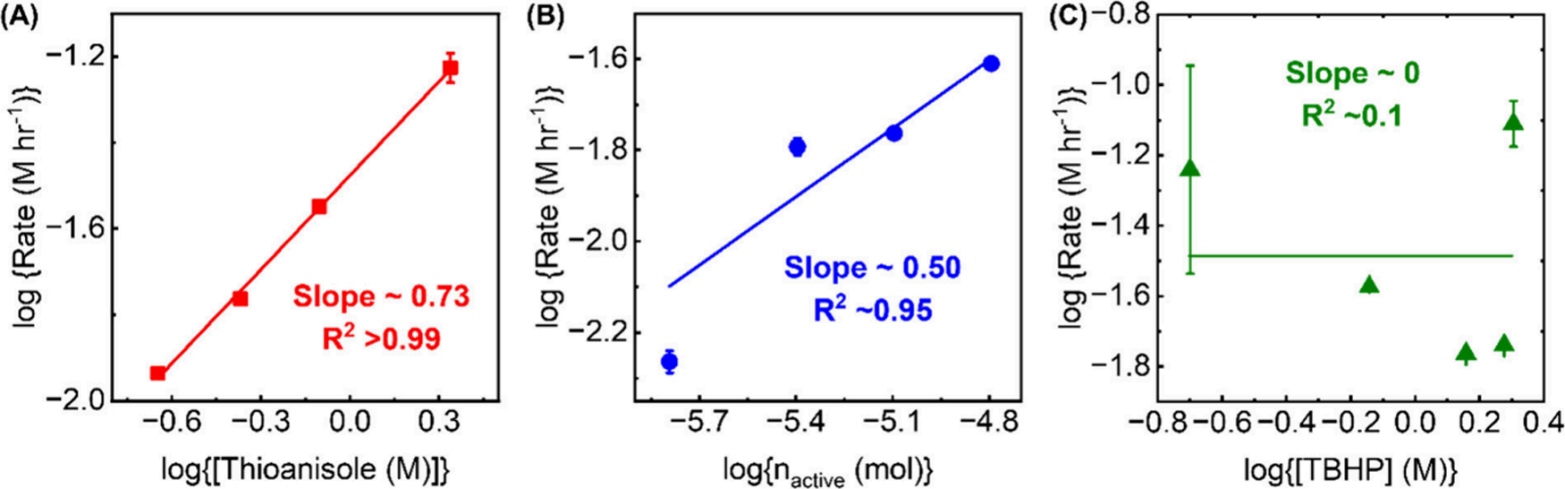
Plots showing log­(rate) vs (A) log­([Thioanisole]), (B) log­(n_active_), or (C) log­([TBHP]). The data points and error bars in (A–C) are the average of at least duplicate measurements of initial rates, determined from the exponential fits (see the SI for more details). In many cases, error bars are smaller than the data points.

Using a similar approach, we have examined how the kinetics are altered upon changing the amount of MOF and the oxidant. An increase in the number of moles of MOFs (n_MOF_) also increased the rate of thioanisole. Every Zr_6_ node of MOF-808 presents six pairs of terminal – OH/OH_2_ groups.^31^ Based on previous findings on ZrO_2_ and other Zr-based MOFs, we believe these are the catalytically responsible sites.^25,38^ Thus, from here onward, we employ the number of moles of active sites (n_active_), which is simply six times n_MOF_. In this case, though scattered, the apparent rate order is roughly 0.5, which again may suggest some diffusive complications (Figure 2B).^35–37^

Finally, the concentration of TBHP was modulated over more than 1 order of magnitude. While TBHP is certainly necessary for product formation (cf.^12,25^ ), the overall reaction rate did not systematically trend with its concentration (Figure 2C). Thus, we conclude that the reaction is in zero order with respect to TBHP concentration. Together, the following rate law is proposed for MOF-808-catalyzed thioanisole oxidation (eq 2).

2 $$\mathrm{Rate}=k{\left[\mathrm{Thioanisole}\right]}^{0.7}{n_{\mathrm{active}}}^{0.5}{\left[\mathrm{TBHP}\right]}^{0}$$

### Modulation of Oxidizing Agent and Addition of a Lewis Acid Inhibitor

Beyond TBHP, H_2_O_2_ is another common sacrificial oxidant used in the oxidation of thioanisole and others.^6,25,33^ When the same reaction was performed using equimolar amounts of H_2_O_2_, *even in the absence of Zr-MOF-808*, H_2_O_2_ was competent in oxidizing thioanisole to the sulfoxide with a conversion of 96(1)% after 48 h (see Figure S7 and the relevant section in the SI for details). The addition of Zr-MOF-808 indeed increased the initial conversion rate, and the major product was again sulfone with 76(5)% selectivity. We suspect that in the presence of a MOF, thioanisole was initially oxidized to sulfoxide (perhaps without the need for a MOF), which was further oxidized to sulfone at the Zr_6_ node. Since this complicates accurate rate determination and kinetic analysis, we prefer to focus on TBHP as an oxidant.

Inhibitors like phenylphosphonic acid (PPA) are commonly introduced into the reaction mixture to validate whether the Lewis acidic sites within a catalyst are responsible for the observed reaction.^33,39,40^ Upon titrating 1, 3, and 6 equivalences of PPA with respect to the moles of MOF into the reaction mixture (see the SI for experimental details), we have indeed observed a systematic decrease in the reaction rate and the conversion; see Figure 3A. The addition of 1 equivalence of PPA should block up to one out of six possible reactive sites per node of Zr-MOF-808 (vide supra). In other words, PPA should *not* influence the immediate surroundings of the five other reactive sites. Yet, as shown in Figure 3B, product selectivity has significantly shifted such that we observe *quantitative selectivity toward sulfoxide*. This is consistent throughout all equivalences examined in this study (Figure S12). Furthermore, when one equivalence of PPA was titrated, the overall conversion reached 64(5)% – in other words, the conversion was *higher* than the reaction without PPA. Even with 6 equivalences of PPA, the MOF is still competent in thioanisole oxidation, reaching 16(5)% conversion; this is well above the background conversion as described earlier. Implications of these observations are discussed later.

**Figure 3:**
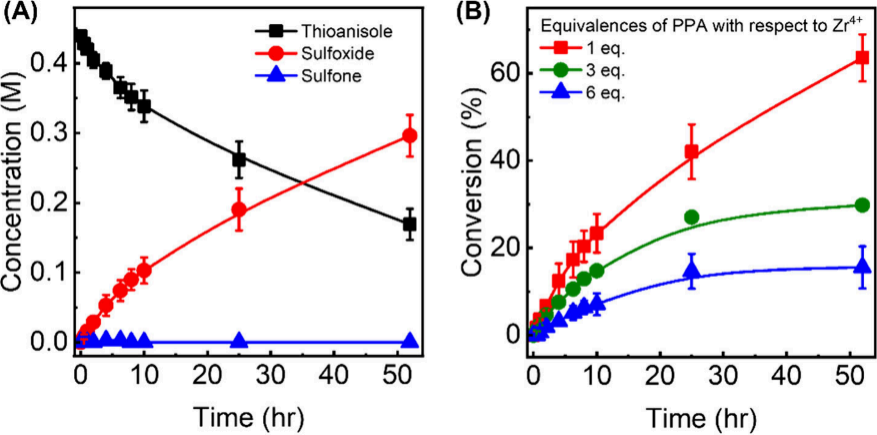
(A) Representative reaction profile of Zr-MOF-808-catalyzed thioanisole oxidation to corresponding sulfoxide and sulfone in the presence of 1 equivalence of PPA. (B) Plot showing conversion of thioanisole vs time in the presence of 1, 3, and 6 equivalences of PPA. All equivalences of PPA are with respect to the amount of Zr cations within the system. The data points and error bars in (A–B) are the average of at least two duplicate measurements.

### Computational Simulations of Reaction Mechanisms

The above experimental observations established that (A) Zr-MOF-808 is catalytically responsible for thioanisole oxidation and (B) its product selectivity depends heavily on the reaction conditions. To understand the reaction mechanism better, we turned to computational simulations to probe the thermodynamics and kinetics of elementary steps.

Figures 4A and B represent the proposed catalytic cycle and free energy profile of thioanisole oxidation first to sulfoxide, and then to sulfone, respectively. Optimized geometries of the first transition state (for the first OAT) and the second transition state (for the second OAT) are presented in Figure 4C. Optimized geometries of all intermediates and transition states are also presented in Figure S14 of the SI. To reduce the computational cost, we replaced the t-butyl group with methyl (represented as R in Figure 4A and as phenyl carboxylate linker with the format) consistent with previous approaches.^5,41–44^ The atomic coordinates of the computed model can be found in the SI.

**Figure 4:**
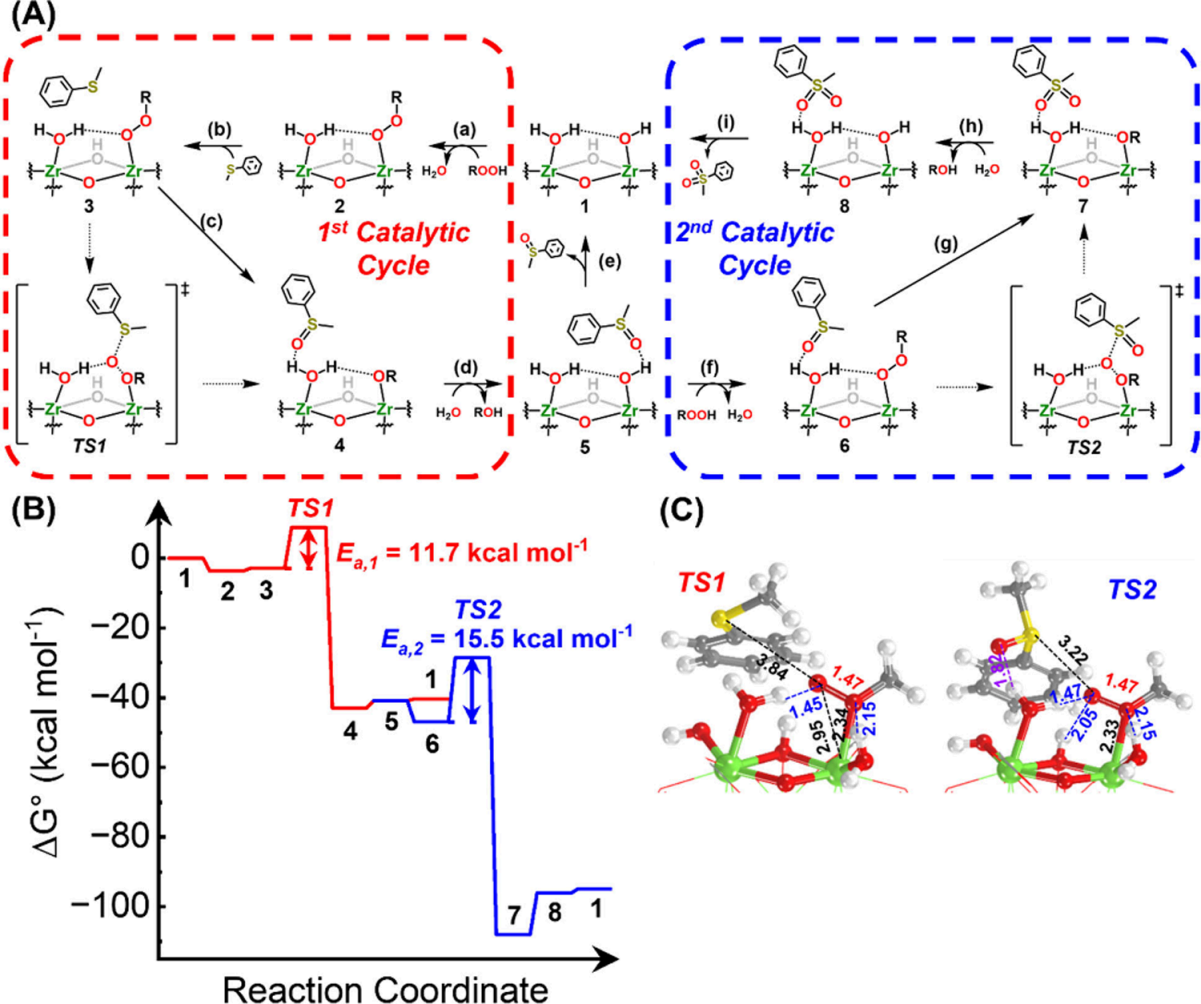
(A) Catalytic cycle of Zr-MOF-808-catalyzed thioanisole oxidation (presented structures (1–8) do not represent optimized structure) and (B) corresponding free energy profile. (C) Computationally predicted, geometry-optimized structures and bond distances of the two transition states. Computed structures and bond distances of those other than the transition states can be found in the SI (distances are shown in Å). H-bonds are shown in blue or purple (for S=O···H–O­(H) interaction). Other relevant atomic distances are shown in black or red (for O–O bond). Atom colors: Green = Zr, Red = O, Gray = C, White = H, Yellow = S.

The catalytic cycle begins with the Zr_6_ node binding to TBHP. Through deprotonation of TBHP, the terminal −OH group initially bound to the Zr^4+^ cation is removed as H_2_O, yielding Zr–OOtBu group (step (a) in Figure 4A). This step is exergonic by ∼3.6 kcal mol^–1^. Note that in this calculation, the solvation of the TBHP and water, as well as partial solvation of the surface intermediates, were not included. At room temperature, this suggests that the molar ratio between bound vs solubilized TBHP is >100:1, and the active sites are likely covered with the −OOtBu species, which provides an explanation for the lack of correlation between the reaction rate and TBHP concentration. In contrast, displacement of the terminal −OH_2_ group with TBHP was overall endergonic by ∼25 kcal mol^–1^, suggesting this reaction is energetically unfavorable under our reaction conditions (see Figure S15 and the SI for more details). When thioanisole approaches the catalytic Zr–OOtBu moiety, the distal ‘OtBu’ functional group at the Zr site is slightly distorted to facilitate nucleophilic attack by thioanisole (denoted TS1 in Figure 4C). The calculated energy profile suggests that this coupling step is the rate-determining step in thioanisole oxidation to sulfoxide, agreeing with our apparent rate law. Namely, only the tBuOO-bound node and thioanisole are involved in the reaction and not the solubilized TBHP. The activation barrier of this OAT reaction was calculated to be roughly 11.7 kcal mol^–1^, suggesting that the reaction is feasible at room temperature. Subsequent desorption of −OtBu group to regenerate the catalytic site was endergonic by 3 kcal mol^–1^_,_ likely due to the H-bonding between the node-bound −OtBu and the adjacent −OH_2_ groups. Computed structures of all relevant intermediates can be found in Figure S14 of the SI.

The desorption of sulfoxide from the node was nearly thermoneutral (stabilized through H-bonding); see steps between 5 and 1 in Figure 4B. In contrast, the introduction of the second TBHP that binds to the sulfoxide-bound node was exergonic by 6 kcal mol^–1^ (step between 5 and 6 in Figure 4B). This energy difference between the desorption of sulfoxide and the adsorption of TBHP suggests that sulfoxide may remain near the node for another oxidation to sulfone. This stability of sulfoxide at the node, combined with the large energy gain of the sequential OAT, enables the further oxidation over Zr-MOF-808, which yields sulfone over sulfoxide in nearly all reaction conditions, despite the higher energetic barrier to oxidize sulfoxide to sulfone (15 kcal mol^–1^; denoted TS2 in Figure 4C). Further elaborations on selectivity vs reaction conditions are described in the Discussion section. Desorption of the doubly oxidized sulfone, like sulfoxide, is nearly thermoneutral; under the reaction conditions, roughly half of the yielded sulfone may likely remain bound to the node, reducing the number of available active sites. This competitive adsorption induced by the sulfone product may explain why, under no reaction conditions, we observed quantitative conversion of thioanisole. We note that the overall reaction mechanism is similar to that proposed for structurally related Zr-based MOF, UiO-66,^25^ though their activity and selectivity were quite distinct; see the Discussion section for more details.

The above calculations were all performed *in vacuo*. To assess the role of solvation, if at all, in reaction energetics, we employed the implicit continuum solvation model with the dielectric constant of 37.5 representing MeCN. As shown in Table S2 and Figure S16, solvation has a negligible effect on the reaction free energies for the first oxidation cycle, with values in the gas phase and in acetonitrile showing only small differences of <2 kcal mol^–1^, which can be attributed to similar solvation energy provided by the solvent to the surface species that cancel out.

Upon the introduction of PPA, the product selectivity shifted quantitatively toward sulfoxide (*vide supra*). We computationally examined the interactions between PPA and the Zr_6_ node. Figure 5A represents the optimized Zr_6_ node in the presence of one PPA molecule (denoted **9** here onward), mimicking the reaction condition with 1 eq. of PPA. Much like other Lewis acidic metal oxides, PPA binding to the Zr_6_ node was thermodynamically downhill by >50 kcal mol^–1^,^33,40^ implying that all PPA molecules within the reaction system should undergo coordination. Subsequent deprotonation of PPA to yield node-bound H_2_O from Zr–OH is unlikely, given that this reaction is instead endergonic by 41.5 kcal mol^–1^ (Figure S17 in the SI). Notably, in the conformation shown in Figure 5A, PPA molecules block many of the terminal/bridging −OH/–OH_2_ groups but leave others open. These sites can coordinate with another PPA molecule when it is introduced, though this reaction is uphill by 6.5 kcal mol^–1^ (Figure 5B and Figure S17); the node with two PPA molecules is denoted as **10** here onward. As discussed below, we believe these interactions, together with the reaction mechanism shown above, directly indicate why the PPA-bound Zr_6_ node prefers to yield sulfoxide quantitatively.

**Figure 5:**
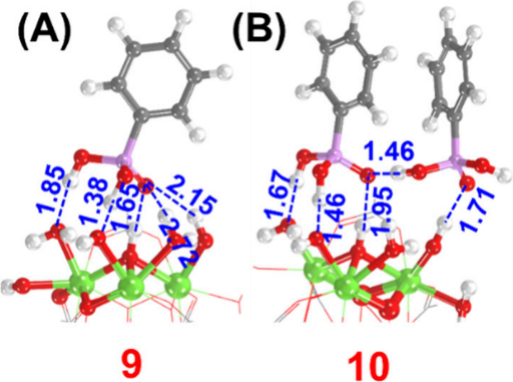
Computed structure of Zr-MOF-808 node bound with (A) one or (B) two PPA molecules. Energetics of these structures and other possible conformations can be found in the SI (distances are shown in Å). All H-bonding interactions are shown in blue. Atom colors: Green = Zr, Red = O, Gray = C, White = H, Pink = P.

## Discussion

### Catalytic Competency of Zr-MOF-808 in Thioanisole Oxidation

Zr-MOF-808 was observed to be catalytically active in thioanisole oxidation. In the presence of TBHP as a sacrificial oxidant and a solvent lacking any labile O-atoms (i.e., in d_3_-MeCN), the major product was, in most cases, the doubly oxidized methyl phenyl sulfone.

The observed catalytic competency of Zr-MOF-808 was surprising, given that another Zr-based MOF, NU-1000, has been reported to be catalytically innocent under exactly identical reaction conditions.^12^ It was the comparison of this finding versus our experimental results that led to our motivation to examine the mechanism, thermodynamics, and kinetics of Zr-MOF-808-catalyzed thioanisole oxidation. NU-1000 has hierarchical pores of 10 and 30 Å, with its node being structurally similar to that of Zr-MOF-808. Thus, the lack of activity toward thioanisole oxidation, at first glance, contradicts our findings using Zr-MOF-808 with smaller pores of 18 Å.^31^

This difference in activity between Zr-MOF-808 and NU-1000 may be due to two reasons. First, Lee et al. removed the modulators of NU-1000 through acid-treatment in dimethylformamide (DMF);^12^ as described by Hupp and co-workers previously, this may induce excessive formation of formate anions due to the thermal decomposition of DMF, displacing the −OH/–OH_2_ groups.^45^ As described in the Results section, the nodes of Zr-MOF-808 have a minimal amount of formate. Thus, the activation of TBHP at the formate-bound node may be hindered kinetically and/or thermodynamically. We note, however, that another Zr-based MOF, UiO-66, with formate/benzoate-bound nodes still presented some catalytic activity toward thioanisole oxidation, though these reactions were performed at elevated temperatures.^25^ Thus, it is tempting to claim that the presence of formate on the node of NU-1000 may not be the only reason for its lack of activity.

Perhaps a more attractive hypothesis for the difference in activity can be ascribed to the distinct microenvironment within the pores of MOF-808 vs NU-1000. The nodes of NU-1000 have two −OH/–OH_2_ groups pointing toward the 30 Å hexagonal channel, while the other two reside within <10 Å pore parallel to the crystallographic *c*-axis (hence often referred to as the c-pore).^46,47^ These −OH/–OH_2_ groups have been demonstrated to be labile, particularly in the presence of coordinating substrates/products.^48,49^ We attribute the apparent lack of activity of NU-1000 to the difference in microenvironment dictated by the MOF pores. Coordination of t-BuOO-group *and* thioanisole (or its oxidized products) on both ends of <10 Å c-pore may be sterically demanding; thus, we expect the reaction to primarily occur at sites pointing toward the 30 Å channel. This one-dimensional hexagonal channel is parallel to the longitudinal axis of the crystals, and thus, access of sterically bulky substrates to these pores solely occurs at the two ends of the crystals.^50–52^ While we are unsure of the exact reason for the lack of catalytic activity of NU-1000, it is conceivable that slow diffusion and coordination of TBHP and thioanisole to the node-activated TBHP may be one of the reasons, though it does not explain the complete lack of activity.

Catalysts residing within the broadly solvent-filled hexagonal channel of NU-1000 tend to have lower activity than those within the c-pores. Previously, Mo­(SH)_2_-functionalized NU-1000 was employed as an electrocatalyst for the reduction of H_2_O to H_2_ in the presence of molecular redox mediators (e.g., methyl viologen).^35,36^ Mo­(SH)_2_ within the hexagonal channel was found to be at least four times *less active* than those within the c-pores. While we acknowledge that the reaction conditions in the H_2_ evolution reaction versus thioanisole oxidation in aprotic *d*_*3*_-MeCN are quite distinct, it is possible that catalysts within broadly solvent-filled, 30 Å hexagonal channels may hinder the substrates, oxidants, and perhaps others from efficiently approaching and coordinating to the catalytic sites. Zr-MOF-808 with more −OH/–OH_2_ groups and with smaller pores of 18 Å may be altogether avoiding these complications, though diffusive ccomplications cannot be removed completely (*vide supra*). Exact validation of these results requires complementary *in-silico* studies that can model the complex motion of solvent, substrates, TBHP, and many others in the pores of MOFs; this remains a challenge given the complexity in accurately modeling the sheer number of atoms involved even within a single MOF pore. Still, MOF-pore-dictated microenvironment and its impact on catalysis have been widely observed. Beyond the electrocatalytic reduction of H_2_O to H_2_ mentioned above, MOF pores dictate the activity and selectivity in C–C bond formation,^53^ hydrogenation,^54^ and many others.^29,55,56^

Another Zr-based MOF, UiO-66, has been reported to be active toward thioanisole oxidation to sulfoxide using H_2_O_2_ as the sacrificial oxidant.^25,38^ While these reports have also observed ‘background’ reactions like that we report here when H_2_O_2_ is employed, the addition of UiO-66 indeed led to an increase in catalytic activity. Furthermore, UiO-66 enriched with ‘missing-linker’ defect sites exhibited higher catalytic activity; this parallels our findings as the nodes of defective UiO-66 present labile – OH/–OH_2_ groups, much like those on Zr-MOF-808. We note that in these reports using UiO-66, methanol was used as the solvent. Thus, the H-bond between sulfoxide and the nodes of Zr-MOF-808, which was the key to high selectivity toward sulfone (*vide infra*), may be disrupted; indeed, for all UiO-66-based catalysis in methanol, sulfoxide was the major product. Our attempts to perform the reported catalysis using TBHP, Zr-MOF-808, and methanol as a solvent failed due to the immiscibility of *n*-decane, the solvent of TBHP. As described above, the use of H_2_O_2_ as a sacrificial oxidant led to 96(1)% conversion after 48 h, precluding accurate kinetic analysis.

### Microenvironment-Governed Sulfoxide vs Sulfone Product Selectivity

Under most reaction conditions examined in this study, Zr-MOF-808 preferentially yielded the doubly oxidized sulfone as the major product over sulfoxide. Even when the TBHP concentration was half of thioanisole (Entry 9 in Table 1), 90% of the observed product was sulfone. This agrees with our computational simulations predicting that it is *thermodynamically more favorable* for TBHP to displace Zr–OH moiety in the presence of proximal sulfoxide by 6 kcal mol^–1^ than for the sulfoxide to undergo desorption. In other words, even when less than one equivalence of TBHP with respect to thioanisole is present, the system has a significant thermodynamic driving force for sulfoxide oxidation to sulfone. Desorption of sulfoxide from the node is nearly thermoneutral, establishing the equilibrium shown in eq 3. Sulfoxide was observed to be the major product within 10 h for many reaction conditions (e.g., Figure 2A), which is likely probing those liberated in the solution. The desorption of sulfone is also thermoneutral, and thus the solubilized sulfoxide can displace sulfone (eq 4). Because TBHP coordination to the sulfoxide-bound node is thermodynamically more favorable than sulfoxide desorption (*vide supra*), the major product is and should be sulfone at the end of the reaction.

3 $$\mathrm{Sulfoxide‐}{\mathrm{Node}}_{\left(s\right)}⇌{\mathrm{Sulfoxide}}_{\left(\mathrm{MeCN}\right)}+{\mathrm{Node}}_{\left(s\right)}$$

4 $$\mathrm{Sulfone‐}{\mathrm{Node}}_{\left(s\right)}+{\mathrm{Sulfoxide}}_{\left(\mathrm{MeCN}\right)}⇌\mathrm{Sulfoxide‐}{\mathrm{Node}}_{\left(s\right)}+{\mathrm{Sulfone}}_{\left(\mathrm{MeCN}\right)}$$

When methyl phenyl sulfoxide was introduced instead as a substrate, Zr-MOF-808 oxidized it to the corresponding sulfone quantitatively within 24 h of the reaction. This contrasts with reactions performed using thioanisole as a substrate, as conversions >80% were never observed even after >48 h of reaction. The difference in free energy of TBHP binding to the pristine node vs that with bound sulfoxide explains this apparent discrepancy in reactivity. According to Figure 4A, TBHP adsorption to the sulfoxide-bound node is *two times more thermodynamically favorable* than that to the pristine node. At a glance, this is counterintuitive because sulfoxide presents more steric hindrance that would increase the energetic penalty of TBHP binding to a proximal Zr^4+^ cation. The H-bonded networks between the two states are nearly identical, except that in the presence of sulfoxide, the S=O group undergoes H-bonding with a nearby Zr–OH_2_ group. We suspect that this withdraws some electron density from the terminal −OH_2_ group, which in turn strengthens the H-bond between the −OH_2_ group and the bound −OOtBu group (shown in a violet dotted line in Figure 4C), further stabilizing the latter.

Sulfoxide was the major product only when (A) thioanisole concentration was >2 M, (B) the mol % of the catalyst was limited to 0.5%, or (C) PPA was present in the reaction mixture. When thioanisole is present in excess, either due to large concentrations of thioanisole or due to the lack of active sites, desorption of sulfoxide should be promoted simply from Le Chatelier’s Principle. Using one equivalence of PPA with respect to the active sites, the overall conversion slightly increased compared to the reaction without PPA, and the selectivity shifted quantitatively toward sulfoxide. The introduction of more PPA led to a significant decrease in activity. This is counterintuitive, given that PPA is an *inhibitor* that should block the active sites. Our computational calculations show that while PPA binding is highly exergonic, agreeing with previous reports.^33,40^ With one molecule per Zr_6_ node, there are many terminal – OH/–OH_2_ groups that can still undergo coordination with TBHP and thioanisole. However, the Zr–OH_2_ groups that the sulfoxide would otherwise undergo H-bonding on the pristine node are blocked by PPA, and thus, the sulfoxide desorption should be preferable. Not only does this explain the selectivity toward sulfoxide, but the slight increase in activity is likely due to PPA preventing sulfoxide from remaining bound on the node, effectively regenerating the active sites and ‘poisoning’ the catalyst toward overoxidation. Introduction of the second PPA molecule completely blocked the catalytic sites for thioanisole oxidation but with the energetic penalty of 6.5 kcal mol^–1^ (Figure 5B), which explains the observed activity *even when six equivalences of PPA per MOF is introduced to the reaction mixture*.

The shift in product selectivity between sulfoxide and sulfone simply through the change in reaction conditions or Lewis acid inhibitor suggests that *Zr-MOF-808-catalyzed sulfone formation is very sensitive to the local microenvironment (particularly H-bonding in stabilizing the surface intermediates) surrounding the active sites*. The complete oxidation of thioethers to sulfones is important to the synthesis of conductive polymers, membranes, organocatalysts, and others.^57,58^ On the other hand, for reactions like the oxidative detoxification of mustard gas (a chemical warfare agent), the selectivity toward singly oxidized and nontoxic sulfoxide is crucial.^59^ Given our observations, it is tempting to claim that one active catalyst may be viable for both oxidative transformations, simply by altering the reaction medium.

## Conclusions

Zr-MOF-808 with Lewis acidic nodes was catalytically competent in thioanisole oxidation in the presence of TBHP as an oxidant. The pristine MOF was observed to be selective toward the doubly oxidized methyl phenyl sulfone in most reaction conditions. This competency was unexpected as related Zr-based MOF, NU-1000, with structurally similar nodes, were incompetent under identical conditions.^12^ We speculate complex interplay of diffusion through pores of different sizes and pore orientations, and the microenvironment dictated by the pore geometry, contributes to this drastic difference in reactivity. Our current interest includes the exploration of other MOFs with distinct pore sizes and investigating how to accurately model such a complex microenvironment to further rationalize and optimize MOF pores for higher activity.

Our findings also indicate that reagents that can competitively coordinate to the active sites and oxidants play equally important roles in dictating the activity and selectivity in the thioanisole oxidation reaction. The introduction of PPA completely changed the selectivity toward sulfoxide. H_2_O_2_ can also serve as an oxidant, though without a MOF, the system was quite active in converting thioanisole to sulfoxide. All of these factors change what would otherwise be called primary and secondary coordination spheres in homogeneous catalysis. We argue that these concepts and theories can be applied to MOF-based catalysts (as their structures are well-defined, like homogeneous catalysts) and should be considered in tuning the catalytic activity and selectivity.

Thioanisole and other thioether oxidation reactions are just one of many OAT reactions that are important in chemical sectors. We believe our findings serve as a cornerstone to highlight how atomically precise catalysts facilitate understanding of the exact chemistry occurring during the reaction and thereby leverage both the catalyst and the surrounding microenvironment to enhance activity and selectivity toward desired products.

## Supplementary Material



## Notes

Additional experimental and computational details and physical characterization (PDF)
