# Supplementary material for: Node-Solution Microenvironment Governs the Selectivity of Thioanisole Oxidation within Catalytic Zr-Based Metal–Organic Framework
Source: ACS Appl Mater Interfaces. 2025 Jul 18;17(30):43112–21. doi: 10.1021/acsami.5c09484 (PMC12314863; doi:10.1021/acsami.5c09484)
Supplement: Supplementary file 1 [file am5c09484_si_001.pdf]

Supporting Information

Node-Solution Microenvironment Governs the Selectivity of Thioanisole Oxidation within Catalytic Zr-based Metal–Organic Framework

Hafsa Abdul Ghuffar,[1] Zaheer Masood,[2] Bin Wang,[2,] and Hyunho Noh[1,*]*

[1]Department of Chemistry and Biochemistry, The University of Oklahoma, Norman, Oklahoma 73019, United States

[2]School of Chemical, Biological and Materials Engineering, University of Oklahoma, Norman, Oklahoma 73019, United States

*Corresponding author

Hyunho Noh: hyunho.noh-1@ou.edu

Table of Contents

1 General Considerations 3
1.1 Materials 3
1.2 Instrumentation 3
2 Physical Characterization of Zr-MOF-808 4
2.1 N_2-Adsorption-Desorption Isotherms 4
2.2 PXRD Patterns 4
2.3 SEM Images 5
2.4 ^{1}H NMR Spectra of Digested Zr-MOF-808 5
3 Details on Catalytic Thioanisole Oxidation 5
3.1 ^{1}H NMR Spectra for Kinetics 5
3.2 Thioanisole Oxidation with and without Zr-MOF-808 using TBHP/H_2O_2 6
3.3 Methyl Phenyl Sulfoxide Oxidation 7
3.4 Thioanisole Oxidation in Various Reaction Conditions 8
3.5 Thioanisole Oxidation in the Presence of PPA 10
3.6 Kinetic Analysis using Single Exponential Fits 11
4 Computational Calculations on Reaction Kinetics 12
4.1 Details on Computational Calculations 12
4.2 Geometry-Optimized Structures of Intermediates and Transition States 13
4.3 XYZ Coordinates 16
5 References 29

1 General Considerations

1.1 Materials

All chemicals and materials in this work were used as received.

The following chemicals were used for the synthesis of Zr-MOF-808. Zirconyl chloride octahydrate ($ZrOCl_2 \cdot 8H_2O$; 98%) and methyl phenyl sulfoxide (98%) were obtained from Fisher Scientific. thioanisole (99%), 1,1,2,2-tetrachloroethane (≥99.0%), 1,3,5-benzenetricarboxylic acid (H_3BTC; 95%), formic acid (≥88%), and *t*-butyl hydroperoxide (5 – 6 M) were purchased from Sigma-Aldrich. Phenylphosphonic acid (PPA; 98%) was purchased from Thermo Scientific.

d_3-MeCN (99.8% D) was purchased from Cambridge Isotope Laboratory. NaOD (40 wt% in D_2O) and D_2O (99.9% D) were purchased from Sigma-Aldrich.

Zr-MOF-808 was synthesized according to the reported procedure.[1]

1.2 Instrumentation

The porosity of Zr-MOF-808 crystallites was confirmed through N_2-adsorption-desorption isotherm using 3Flex (Micromeritics). The freshly synthesized sample was thermally activated under a dynamic vacuum (<50 mTorr) at 120 °C using VacPrep (Micromeritics) overnight, following the reported procedure. Using the data set within the P/P_0 range of 0.005 – 0.1,[2] the Brunauer –Emmett–Teller (BET) area of Zr-MOF-808 was calculated to be ….. m^2/g, which agrees well with other reports.[1,3] The isotherm and the DFT-derived pore size distribution are shown below.

The powder X-ray diffraction (PXRD) patterns of Zr-MOF-808 before and after catalysis were measured using Rigaku Smartlab equipped with a Cu Kα X-ray source. 2θ between 2 – 50° with a step size of 0.01°/min were employed as parameters. The background signal was removed using Origin.

Scanning electron microscopy (SEM) images of Zr-MOF-808 were collected using the Zeiss Neon 40 EsB field emission instrument operated at 15 kV. Briefly, the suspension of Zr-MOF-808 in acetone was drop-casted onto a polished Si wafer and was air-dried. The wafer was supported on the conventional SEM sample holder using carbon tape. Using EMS Quorum Q160 ES plus sputter coater, around 4 nm of iridium was coated.

^1H NMR spectra (Varian VNMRS 400 MHz) were used to measure the concentration of reactants and products; see below for experimental details.

2 Physical Characterization of Zr-MOF-808

2.1 N_2-Adsorption-Desorption Isotherms

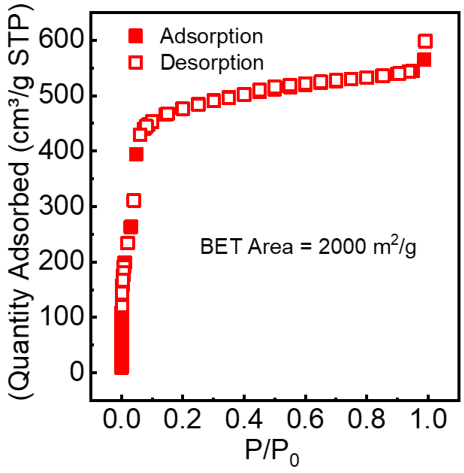

Figure S1. N_2-adsorption-desorption isotherm of Zr-MOF-808.

2.2 PXRD Patterns

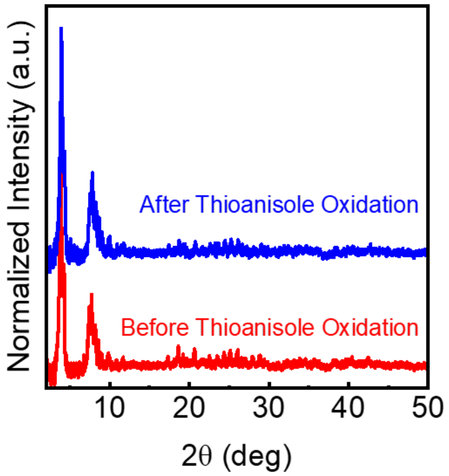

Figure S2. PXRD patterns of Zr-MOF-808 before and after thioanisole oxidation.

2.3 SEM Images

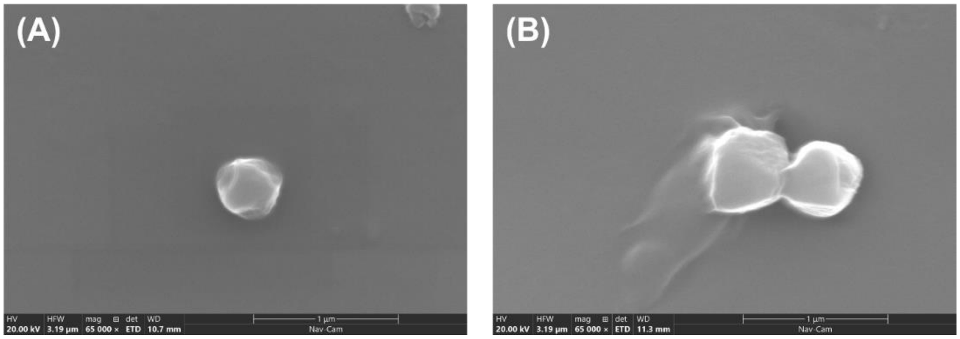

Figure S3. SEM images of Zr-MOF-808 (**A**) before and (**B**) after thioanisole oxidation.

2.4 ¹H NMR Spectra of Digested Zr-MOF-808

Zr-MOF-808 was synthesized using formic acid as a modulator and subsequently treated with 1 M HCl(aq) to remove the formate units from the node. To confirm that the node-bound formate units have been removed, ^{1}H NMR spectra of Zr-MOF-808 digested in ~1 M NaOD in D_2O before and after acid activation were measured; see Figure S4. The peaks at δ = 8.08 and 8.03 ppm correspond to H's on formate or BTC^{3-}, respectively. Based on the integration ratio of the two peaks, we concluded that the nodes of Zr-MOF-808 before and after the 1 M HCl treatment have ~4 or ~0.5 formates/Zr_6 node. Thus, Zr-MOF-808 employed in catalysis have minimal amount of formate units remaining on the node.

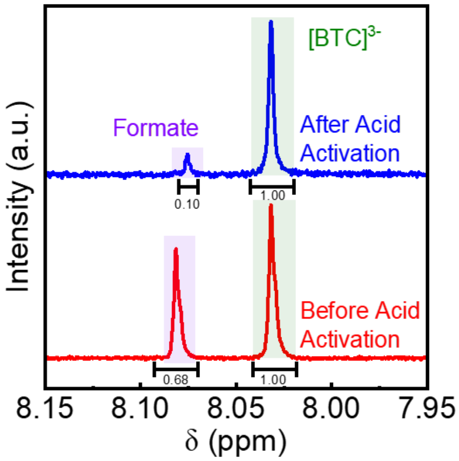

Figure S4. ^{1}H NMR spectra of digested Zr-MOF-808 before and after acid activation in 1 M $HCl_{(aq)}$.

3 Details on Catalytic Thioanisole Oxidation

3.1 ¹H NMR Spectra for Kinetics

Below are representative ^{1}H NMR spectra of catalytic thioanisole oxidation in the presence of Zr-MOF-808. Similar ^{1}H NMR spectra were observed for all reaction conditions. Features assigned to thioanisole, sulfoxide, and sulfone are probing the protons on the -CH_3 group of the molecules.

Internal standard (IS) refers to the two protons on 1,1,2,2-tetrachloroethane. Integrations of these peaks were employed to determine the kinetic profiles shown in the next section.

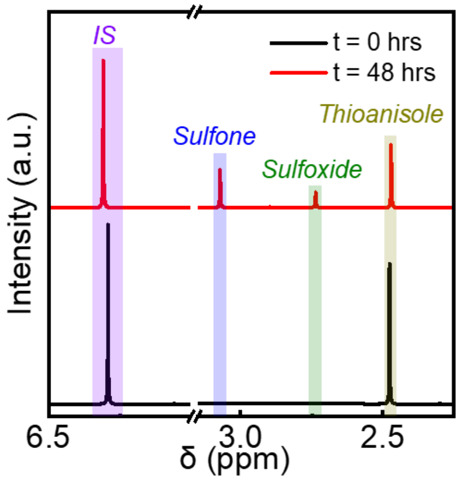

Figure S5. Representative ^{1}H NMR spectra at t = 0 (black) and t = 48 hours of the reaction.

3.2 Thioanisole Oxidation with and without Zr-MOF-808 using TBHP/H_2O_2

Below are kinetic profiles of thioanisole oxidation reaction with and without Zr-MOF-808 introduced as the heterogeneous catalysts. TBHP or H_2O_2 was used as the sacrificial oxidant. When TBHP was used as the oxidant in the absence of MOF, conversion of thioanisole reached only up to 5(2)%, with quantitative selectivity towards sulfoxide after 48 hours of reaction (Figure S5A). This contrasts with 47(1)% conversion at the same time in the presence of MOF, with the major product being the sulfone (Figure S5B); we note Figure S5B is also reproduced as Figure 1A in the main text.

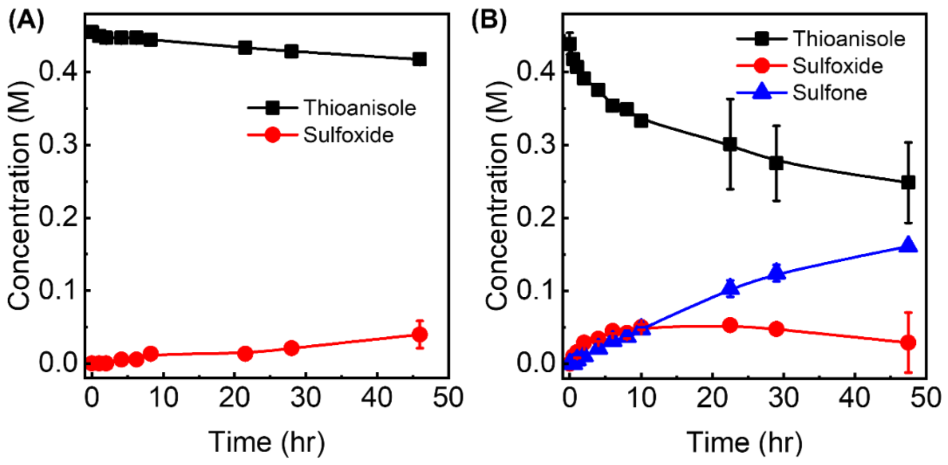

Figure S6. Kinetic profile of thioanisole oxidation using TBHP as the sacrificial oxidant (**A**) without or (**B**) with Zr-MOF-808 in the reaction mixture.

On the other hand, when H_2O_2 was used as a sacrificial oxidant, we observed near quantitative conversion of thioanisole to its corresponding sulfoxide (Figure S6A). In a similar reaction with MOF, the product selectivity shifted towards sulfone (Figure S6B). Large 'background' reaction in the presence of H_2O_2, however, discouraged any kinetic analysis, and thus were not pursued further in this study.

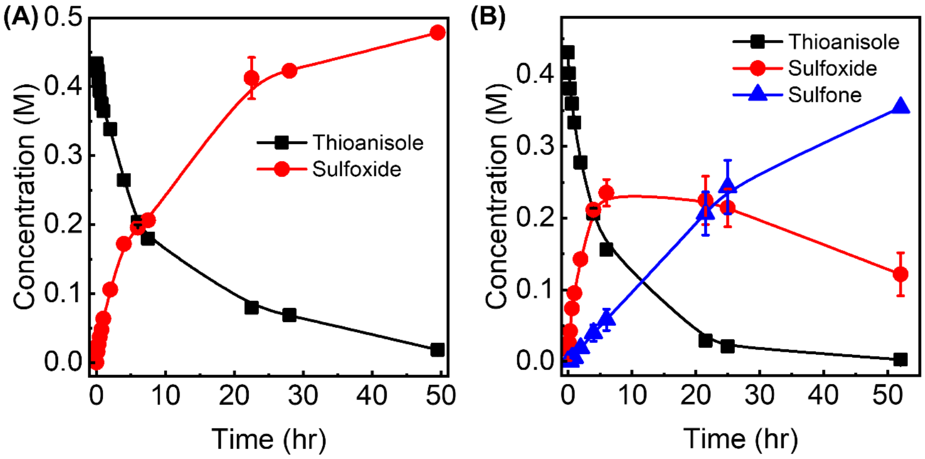

Figure S7. Kinetic profile of thioanisole oxidation using H_2O_2 as the sacrificial oxidant (**A**) without or (**B**) with Zr-MOF-808 in the reaction mixture.

3.3 Methyl Phenyl Sulfoxide Oxidation

Regardless of the oxidant, Zr-MOF-808 oxidized thioanisole primarily to methyl phenyl sulfone. To ensure that this product selectivity is due to the presence of the MOF, methyl phenyl sulfoxide was instead employed as a substrate. As shown in Figure S7, indeed, Zr-MOF-808 converted nearly all of the sulfoxide to sulfone within 25 hours. This reactivity was absent when Zr-MOF-808 was removed from the reaction mixture.

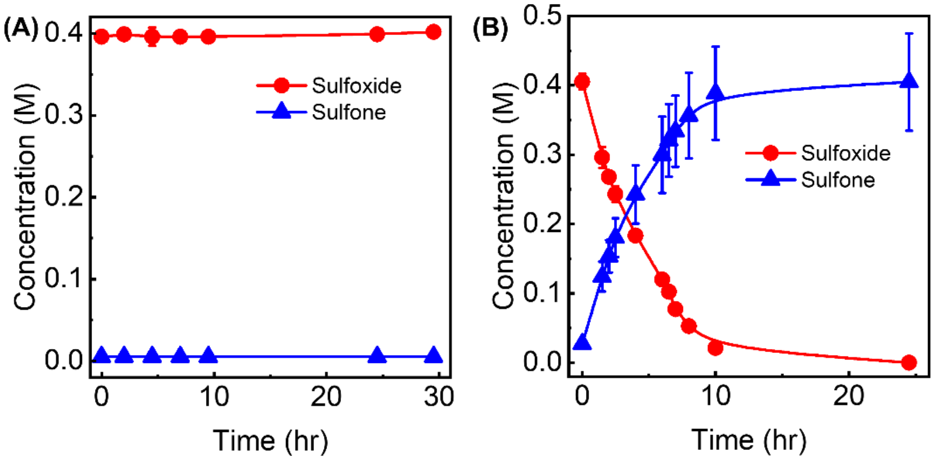

Figure S8. Methyl phenyl sulfoxide oxidation (**A**) without or (**B**) with Zr-MOF-808.

3.4 Thioanisole Oxidation in Various Reaction Conditions

As described in the main text, concentrations of thioanisole, TBHP, and the amount of MOFs were modulated to determine the apparent rate law. The following figures show the kinetic profiles under these reaction conditions.

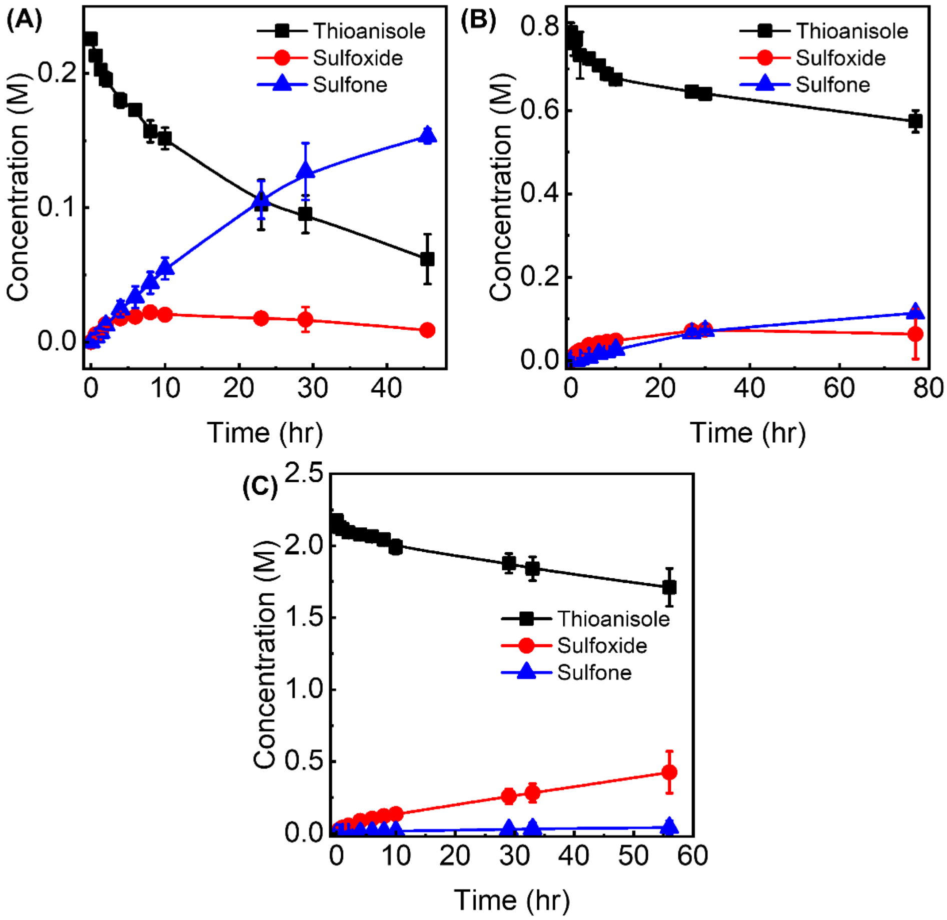

Figure S9. Catalytic profile in the presence of various concentrations of thioanisole. Thioansiole concentrations for individual figures are: (**A**) 0.23, (**B**) 0.79, and (**C**) 2.18 M.

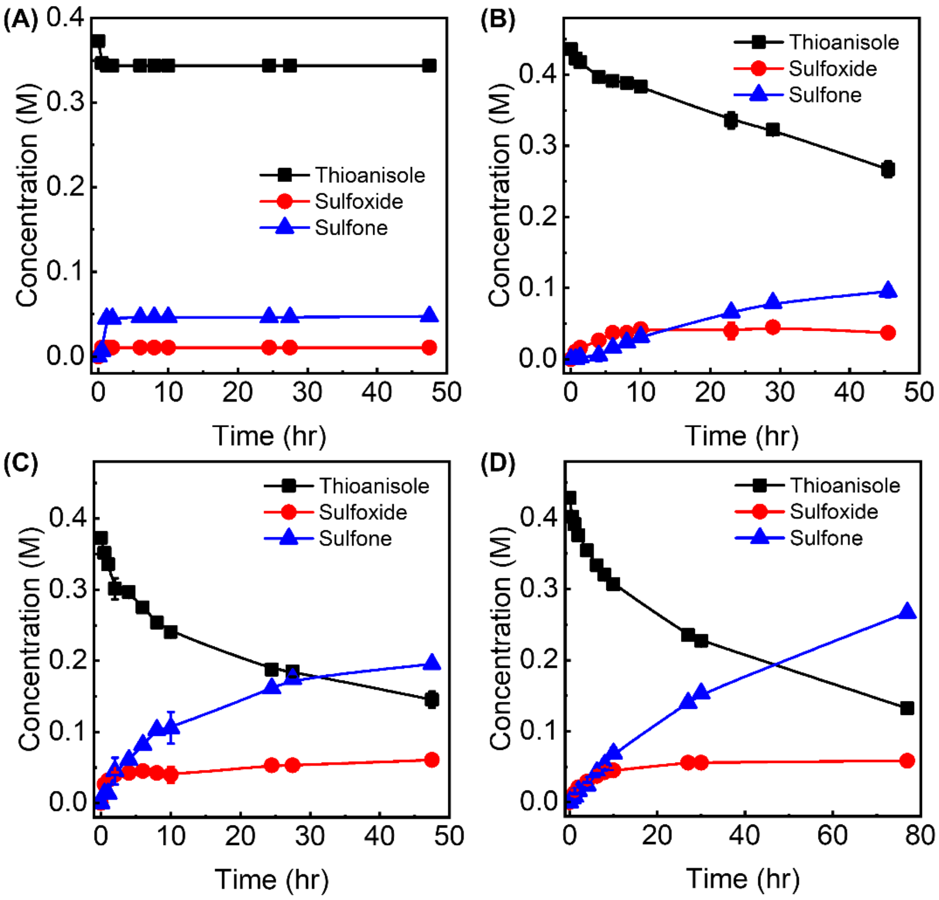

Figure S10. Catalytic profile in the presence of various concentrations of TBHP. TBHP concentrations for individual figures are: (**A**) 0.2, (**B**) 0.7, (**C**) 1.9, and (**D**) 2.2 M.

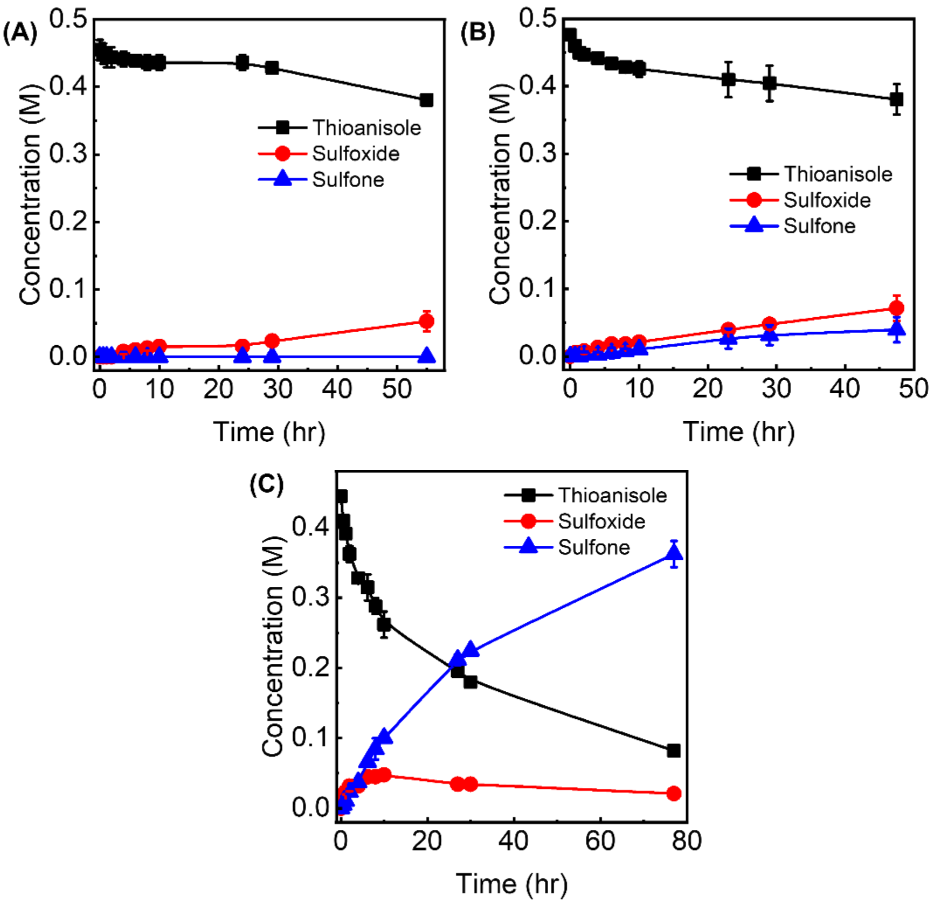

Figure S11. Catalytic profile in the presence of various amounts of Zr-MOF-808. The MOF mol% values for individual figures are: (**A**) 0.5, (**B**) 1.3, and (**C**) 5.2%. For (**A**) sulfone was not detected.

3.5 Thioanisole Oxidation in the Presence of PPA

PPA is a common Lewis acid inhibitor introduced into the reaction mixture to validate that the Lewis acidic sites within the catalysts are indeed responsible for the catalytic reaction. Figure S10 below shows the kinetic profile in the presence of one, three, and six equivalences of PPA with respect to the MOF active sites. We note that the figure with one equivalence of PPA is a replicate of Figure 3A in the main text.

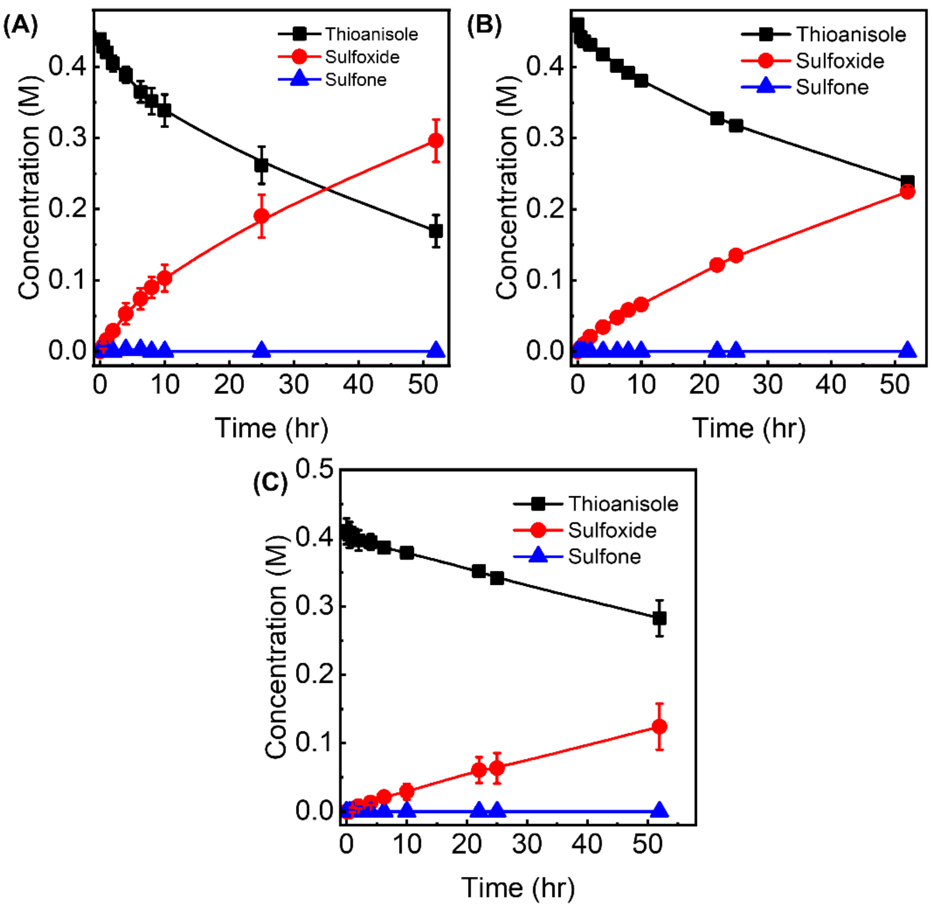

Figure S12. Catalytic profile in the presence of various equivalences of PPA. The PPA equivalences for individual figures are: (**A**) 1, (**B**) 3, and (**C**) 6 equivalences with respect to the amount of active sites. For all reactions, no sulfone was detected.

3.6 Kinetic Analysis using Single Exponential Fits

As described in the main text, the first-order derivative of the single exponential fit that modeled the experimentally observed conversion of thioanisole was used to derive the reaction rates. As described in the following reference,[4] in general, this minimizes diffusive complications, though our rate law does still indicate some diffusive contributions (see the main text). Below is the figure showing the exponential fit of the reaction shown in Figure 1A and S6B.

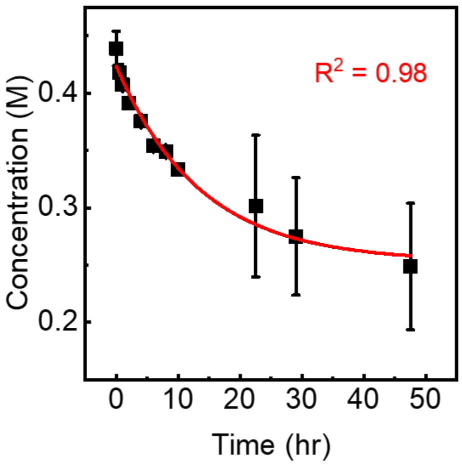

Figure S13. Catalytic conversion of thioanisole with the exponential fit.

The average of the first-order derivatives within the first 30 minutes of the reaction was used to determine the catalytic rate. As shown below in Table S1, changing this time range of 30 minutes to 2 hours did not alter the rate significantly.

Table S1. Rate of thioanisole conversion based on the first-order derivative of exponential fits shown in Figure S12.

Range of Time used for Average (hr)	Catalytic Rate (mM hr^{-1})
0.5	17(1)
1	17(1)
2	16(1)

4 Computational Calculations on Reaction Kinetics

4.1 Details on Computational Calculations

All DFT calculations were performed using the Vienna ab-initio simulation package (VASP).[5] We used the Perdew-Burke-Ernzerhof (PBE) exchange and correlation functional[6] with projector augmented wave (PAW).[7,8] The cell with dimensions a= 25 Å, b = 25 Å, c = 25 Å was used using a single Γ point of Brillouin zone with a planewave kinetic cutoff energy of 400 eV. Zr-MOF-808 node was taken from cif file of Zr-MOF-808, with carboxylate capped with H.[3,9] All structures were optimized until the forces of each atom were converged to less than 0.02 eV $Å^{-1}$. The electronic self-consistency cycles were converged to < 10-6 eV with Gaussian smearing of 0.02 eV. The van der Waals interactions were included using the Grimme dispersion (DFT-D3) correction (IVDW = 11) with zero damping function.[10,11] Transition state calculations were carried out using the climbing image nudged elastic band (NEB)[12] and dimer method.[13] Frequency calculations were

done to confirm transition state and vibrational analysis. True transition state was confirmed by a single imaginary frequency along reaction coordinate.

Electronic energies (E°_{elect}) were calculated from DFT calculations and were converted to free energies for all intermediates using the expression, $G^{\circ} = E^{\circ}_{elect} - TS + ZPE$. TS (T = 298 K). ZPE (zero-point energy) and TS terms were calculated from the vibrational analysis of the adsorbed, and gaseous species at the standard states. The VASPKIT package[14] was used to extract ZPE and TS contribution from frequency calculations. In frequency calculations of adsorbed states, two Zr atoms, one OH and H_2O on each Zr atom and all atoms in adsorbates were allowed to move. Other atoms in node were kept frozen at their optimized positions. We assumed that entropic contributions from the free-rotor approximation[15] are negligible as we demonstrated in our previous study.[16] The energies of all intermediates were also checked by spin polarized calculations and were found comparable to non-spin polarized calculations.

4.2 Geometry-Optimized Structures of Intermediates and Transition States

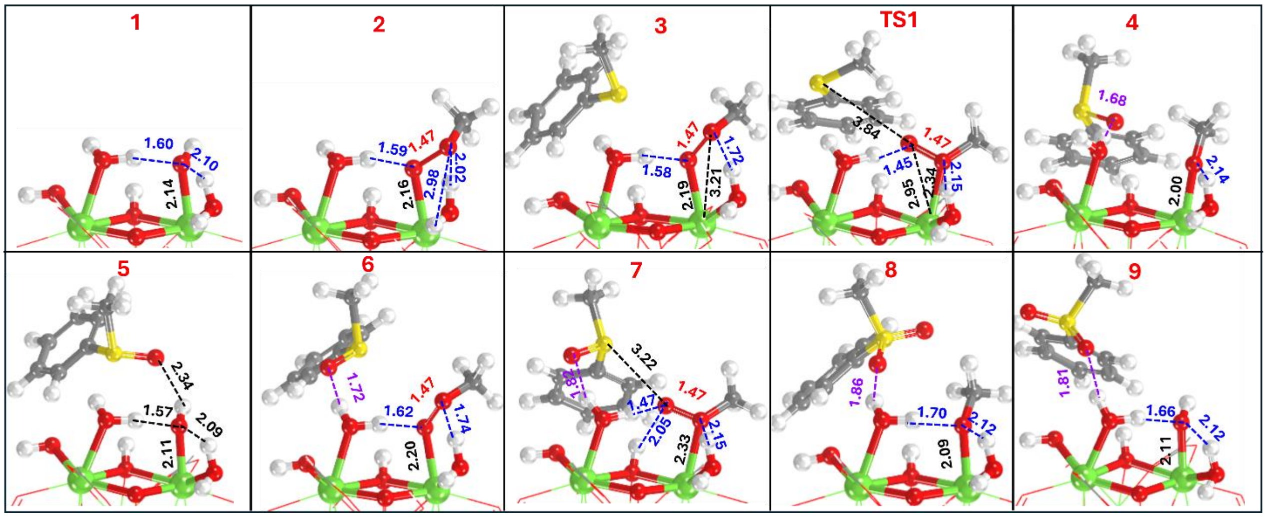

Figure S14. Geometry-optimized structures of intermediate structures in Zr-MOF-808-catalyzed thioanisole oxidation to sulfoxide and sulfone. H-bonds are shown in blue or purple (for S=O···H–O(H) interaction). Other relevant atomic distances are shown in black or red (for O–O bond). Atom colors: Green = Zr, Red = O, Grey = C, White = H, Yellow = S.

In step 2, TBHP displaces Zr–OH group to yield Zr–OOtBu and H_2O and this step is exergonic by ~3.6 kcal mol^{-1}. As shown in Figure S14A, displacement of Zr–OH_2 group by TBHP is endergonic by 25 kcal mol^{-1} and hence is likely not occurring in the reaction mixture; Figure S14B is the geometry-optimized structure of this unstable species. This intermediate is referred to as **11** here onwards.

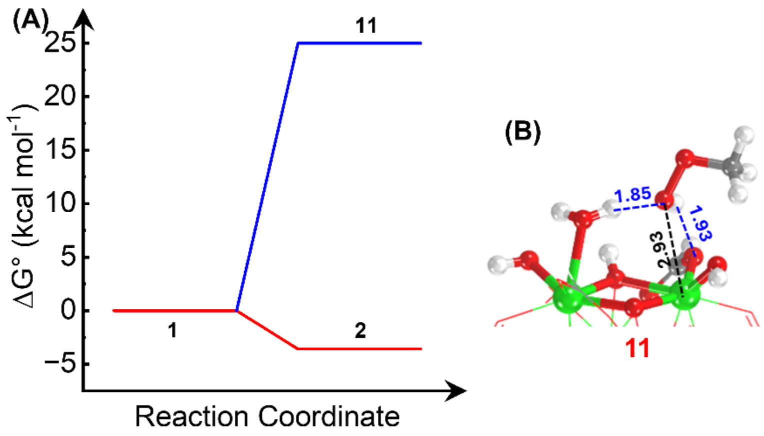

Figure S15. (**A**) Free energy profile of TBHP displacement of Zr–OH or Zr–OH_2 group. (**B**) geometry-optimized structure of TBHP bound to Zr cation that originally was coordinated to –OH_2 group. H-bonds are shown in blue. Other relevant atomic distances are shown in black. Atom colors: Green = Zr, Red = O, Grey = C, White = H, Yellow = S.

To understand the role of solvation, we have computed the energetics of the first catalytic cycle (*i.e.,* conversion of thioanisole to the corresponding sulfoxide) with an implicit continuum solvation model implemented in VASPsol,[17,18] using a dielectric constant of 37.5 to represent MeCN. As shown below in Table S2 and Figure S16, the effect of solvation is minimal with differences in energetics in gas-phase vs. that in MeCN of <2 kcal mol^{-1}.

In Table S2 and Figure S16, step labels and numbers refer to those listed in Figure 4A of the main text.

Table S2. Reaction free energy calculated according to Figure 4A in gas-phase and in MeCN.

**Step Label**	**Reaction Energetics (kcal mol^{-1})**	
	**Gas phase**	**In MeCN**
(a)	–3.61	–2.31
(b)	0.72	1.65
TS, E_a	11.71	11.02
(c)	–40.08	–42.61
(d)	2.08	3.92
(e)	1.12	–0.50

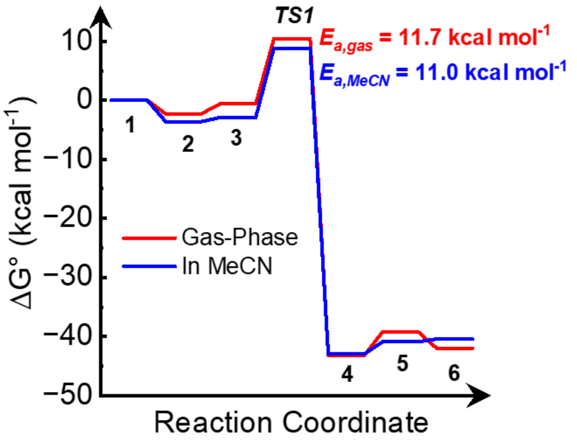

Figure S16. Free energy profile of Zr-MOF-808 catalyzed thioanisole oxidation to sulfoxide with TBHP as a sacrificial oxidant in gas-phase vs. in MeCN.

Thermodynamics of PPA coordination to Zr_6 node of Zr-MOF-808 was also considered. As described in the main text and in Figure S15, coordination of one PPA molecule to the pristine node was thermodynamically downhill (*i.e.,* formation of the intermediate, **9**; see Figure 5A for the computed structure). Coordination of another PPA molecule (**10**) or PPA deprotonation by Zr–OH (**12**) were endergonic, shown by the red or the blue free energy profile in Figure S15, respectively. This suggests why the observed catalytic conversion above background even in the presence of six equivalences of PPA.

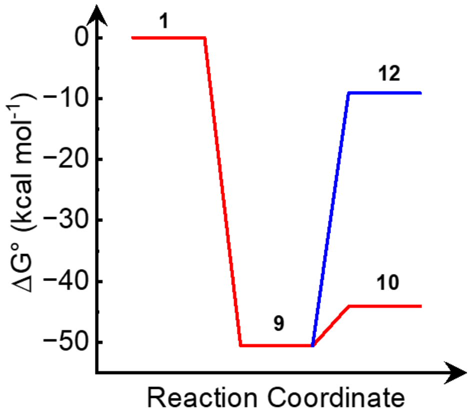

Figure S17. Free energy profile of one or two PPA molecules undergoing coordination with Zr_6 node followed by its deprotonation.

4.3 XYZ Coordinates

The following shows the XYZ coordinates of all computed structures. The label corresponds to that shown in Figures S13-S15, and Figures 4-5 in the main text.

1

1.0

25.0000000000	0.0000000000	0.0000000000
0.0000000000	25.0000000000	0.0000000000
0.0000000000	0.0000000000	25.0000000000

C O Zr H

6 32 6 28

Direct

0.316560000	0.339478999	0.456093013
0.372359008	0.425922990	0.301297992
0.319240004	0.524980009	0.449173987
0.631667972	0.342016011	0.457935989
0.633437991	0.515008986	0.453606009
0.581870019	0.432154000	0.596548021
0.381374002	0.381067991	0.323406994
0.349148005	0.325850010	0.419553995
0.441722006	0.274771988	0.400723994
0.384629995	0.366699994	0.577187002
0.478798002	0.320796013	0.577593029
0.439105004	0.369937003	0.476915002
0.321428001	0.377344996	0.489618987
0.385724008	0.472086996	0.317847997
0.380840003	0.430070996	0.418256015
0.441623986	0.590116978	0.419746995
0.350414008	0.532023013	0.409680992
0.474790990	0.429356992	0.378039986
0.465474010	0.533043981	0.581721008
0.377676010	0.475477010	0.579819024
0.439653993	0.490720004	0.475827992
0.324777991	0.492413998	0.487794995
0.471758991	0.430752993	0.550755978
0.478619009	0.333247006	0.316507995
0.570424020	0.383424997	0.324063003
0.519083023	0.348313987	0.418917000
0.625847995	0.374278992	0.419223011
0.567821980	0.386202008	0.580232024
0.597470999	0.329340011	0.493339002
0.498647004	0.269870996	0.488772988
0.559098005	0.487192988	0.321099997
0.468535990	0.539973021	0.326797992
0.519737005	0.510815024	0.417629004
0.625742018	0.482317001	0.415459991
0.567116976	0.477412999	0.578746021
0.542995989	0.429894000	0.476583987
0.506705999	0.585628986	0.501695991
0.600643992	0.528895020	0.489744008
0.431450009	0.358729988	0.394344985
0.432323009	0.500059009	0.392268986
0.390493006	0.431347996	0.508485019
0.515133977	0.359073997	0.510380030
0.556921005	0.429634005	0.394547999
0.515537977	0.501033008	0.509890974
0.279163986	0.315741003	0.458934993
0.407604009	0.256316006	0.404345006
0.420051992	0.346217990	0.581250012
0.379303992	0.388249010	0.609291971
0.474249989	0.266701996	0.455877006
0.471590012	0.431322008	0.589621007
0.349803001	0.424562007	0.263087988
0.282622010	0.550050020	0.449782997
0.447385013	0.536723971	0.294243008
0.345559001	0.497435004	0.577863991
0.428604007	0.515011013	0.583611012
0.671191990	0.322046012	0.461113989
0.515964985	0.350313008	0.315375000
0.483011007	0.294703007	0.320755988
0.601925015	0.360624999	0.323873997
0.497996986	0.323175997	0.611334980
0.478801996	0.259119004	0.520745993
0.673537016	0.533999979	0.455154985
0.467072994	0.593294978	0.451849997
0.458561003	0.607232988	0.389043003
0.524146974	0.508504987	0.317685008
0.561058998	0.460817993	0.292259991
0.538366020	0.542276025	0.404446989
0.610089004	0.433012009	0.630818009
0.539476991	0.606181026	0.497750014
0.461297989	0.570322990	0.570550978
0.344294995	0.430117995	0.405131012
0.537370980	0.316339999	0.406569004

2

```
1.0
   25.0000000000    0.0000000000    0.0000000000
    0.0000000000   25.0000000000    0.0000000000
    0.0000000000    0.0000000000   25.0000000000
   C   O  Zr   H
   7  33   6  30
Direct
  0.315811008  0.339477986  0.451169014
  0.373329014  0.428003013  0.299169987
  0.318758994  0.525223017  0.448217005
  0.631363988  0.341984004  0.456068009
  0.632605016  0.515161991  0.453817993
  0.582621992  0.433196992  0.594377995
  0.520175993  0.279256999  0.644209027
  0.476110011  0.329715014  0.580012023
  0.496919006  0.276077002  0.592341006
  0.382070005  0.382838011  0.320701987
  0.349236995  0.326043993  0.415291995
  0.442366987  0.275330991  0.398615003
  0.379673004  0.365334004  0.574261010
  0.438843995  0.370097011  0.474482000
  0.320111990  0.376924008  0.485201001
  0.386444002  0.473960012  0.316540003
  0.380654007  0.430536002  0.416121989
  0.440872014  0.590834022  0.420500010
  0.350118011  0.532643020  0.408926994
  0.474741995  0.430415004  0.376387000
  0.463003010  0.531121016  0.581452012
  0.375705004  0.473520994  0.578253984
  0.438731998  0.490245014  0.475270003
  0.323823005  0.491847008  0.486182988
  0.470497012  0.428541005  0.550107002
  0.478953004  0.334499002  0.313888013
  0.570599973  0.384930015  0.322573006
  0.519083977  0.349121004  0.416860998
  0.625639021  0.374879986  0.417874008
  0.570972979  0.387367994  0.576461017
  0.597211003  0.328967988  0.491355985
  0.500066996  0.271129996  0.485567987
  0.559409976  0.488994986  0.320894986
  0.468816012  0.542098999  0.327015996
  0.519093990  0.511572003  0.417512000
  0.625126004  0.482764006  0.415354013
  0.564840019  0.478240997  0.578579009
  0.541776001  0.430103004  0.475528985
  0.505802989  0.585319996  0.502654016
  0.599633992  0.528810024  0.489843011
  0.431659997  0.359479010  0.391984999
  0.432168007  0.500954986  0.391593009
  0.389342010  0.430269003  0.506264985
  0.515249014  0.359263003  0.509019017
  0.556608021  0.430426002  0.393492013
  0.514120996  0.500862002  0.509804010
  0.278198987  0.315955013  0.452692986
  0.408230990  0.256844997  0.401749015
  0.414384991  0.344525993  0.579994023
  0.373921990  0.387432009  0.605958998
  0.474496007  0.267556012  0.453496009
  0.470954001  0.427536011  0.589006007
  0.351186007  0.427222997  0.260711998
  0.282427013  0.550709009  0.449160010
  0.448022991  0.539376020  0.294176012
  0.343789995  0.495727986  0.575845003
  0.426340014  0.512449026  0.582961977
  0.670742989  0.321680009  0.458783001
  0.516233027  0.351790994  0.313255012
  0.483518988  0.295935988  0.317685008
  0.601745009  0.361651003  0.323244989
  0.487408012  0.250611991  0.516309023
  0.672756016  0.534020007  0.455664009
  0.466300011  0.593768001  0.452616006
  0.457648993  0.608466983  0.389995992
  0.524564028  0.510511994  0.317703992
  0.561554015  0.463319987  0.291447997
  0.537600994  0.543250978  0.404695988
  0.611401975  0.434671998  0.628148019
  0.538857996  0.605412006  0.498676986
  0.458074987  0.568699002  0.571821988
  0.344246000  0.430756003  0.402644008
  0.537302971  0.317048013  0.404626012
  0.551142991  0.310555011  0.645601988
  0.489308000  0.286855012  0.674798012
  0.538426995  0.239730000  0.650770009
```

3

1.0

25.0000000000	0.0000000000	0.0000000000
0.0000000000	25.0000000000	0.0000000000
0.0000000000	0.0000000000	25.0000000000

C	O	Zr	H	S
14	33	6	38	1

Direct

0.319599986	0.331775993	0.446108013
0.375331014	0.426165998	0.296306998
0.316069007	0.517809987	0.446274012
0.634844005	0.344606996	0.453395993
0.630524993	0.517521977	0.455612987
0.580536008	0.428317010	0.593699992
0.513471007	0.244783998	0.617200017
0.435658008	0.480580002	0.812460005
0.435618997	0.431205004	0.786472976
0.415764004	0.427148998	0.734144986
0.396917999	0.472808987	0.707700014
0.397965014	0.522288024	0.733684003
0.417044997	0.526250005	0.786023974
0.363934010	0.328857988	0.734156013
0.477923006	0.318740994	0.573826015
0.474175006	0.260214001	0.578716993
0.385010988	0.380793005	0.317005008
0.353531003	0.320522010	0.409960002
0.448852986	0.273564994	0.392311007
0.382183999	0.358803004	0.570239007
0.440806001	0.366071999	0.470627010
0.322263002	0.368640989	0.480939001
0.386963010	0.472043991	0.314842999
0.381503999	0.426041991	0.413663000
0.436787993	0.587588012	0.422221988
0.347923994	0.527320027	0.407880008
0.476020008	0.429710001	0.374518991
0.460509986	0.525379002	0.581915975
0.373632997	0.468820989	0.574670017
0.437487006	0.485893995	0.474647999
0.321628004	0.483815998	0.483642995
0.470081002	0.422861010	0.548377991
0.483880997	0.335792989	0.309749007
0.573876977	0.388900012	0.320318013
0.522594988	0.349023998	0.413255990
0.628256977	0.378008991	0.415749997
0.569374025	0.382862002	0.574513018
0.600709021	0.329315990	0.487740010
0.506110013	0.268696010	0.480399013
0.559198976	0.492352992	0.321119994
0.467117012	0.542177975	0.327919990
0.517563999	0.511080027	0.417991012
0.624466002	0.486016005	0.416175991
0.563090980	0.473710001	0.578508019
0.542313993	0.428846002	0.474094987
0.501136005	0.582190990	0.504621029
0.596817970	0.528963983	0.491735995
0.434866011	0.357266009	0.387946010
0.431160003	0.498371005	0.391281992
0.389099002	0.423974991	0.504225016
0.516924977	0.355980009	0.504616022
0.557814002	0.432063997	0.392293990
0.512275994	0.497505009	0.510109007
0.283044994	0.306603014	0.447203010
0.415580004	0.253499001	0.395155996
0.417636991	0.338297993	0.572202027
0.378477007	0.374181002	0.606265008
0.481770992	0.265486002	0.447503000
0.469599009	0.421627998	0.587296009
0.353794992	0.425603986	0.257479012
0.278764993	0.541868985	0.447005004
0.446494013	0.539340973	0.294988990
0.342016995	0.490931988	0.568983018
0.423617005	0.506850004	0.581396997
0.675116003	0.326146990	0.456445009
0.520559013	0.354250014	0.309740007
0.489657998	0.297342986	0.313113987
0.605741978	0.366607994	0.320847005
0.489241987	0.251271993	0.511952996
0.669993997	0.537676990	0.458386004
0.461906999	0.590458989	0.454638004
0.453307003	0.606334984	0.392248005
0.523651004	0.512772977	0.318147987
0.562552989	0.467480987	0.291096985
0.534976006	0.543703973	0.406035990
0.608555019	0.428995013	0.628120005
0.533677995	0.603199005	0.501199007
0.456265986	0.563049018	0.572192013
0.345223993	0.425421000	0.399870008
0.541893005	0.317802012	0.400483996
0.554170012	0.254696012	0.603336990
0.505524993	0.264530003	0.655775011
0.508867025	0.201137006	0.620985985
0.450893015	0.483581007	0.853287995
0.451397985	0.395711988	0.806461990
0.381980002	0.469395995	0.666752994
0.383662999	0.557919979	0.712795973
0.417661011	0.564958990	0.806281984
0.325412005	0.349344999	0.729362011
0.373223007	0.324225992	0.776820004
0.361681998	0.289090008	0.715613008
0.416904986	0.364818990	0.699769974

TS1

1.0

25.0000000000	0.0000000000	0.0000000000
0.0000000000	25.0000000000	0.0000000000
0.0000000000	0.0000000000	25.0000000000

C	H	O	S	Zr
14	38	33	1	6

Direct

0.319255710	0.333033770	0.449244887
0.383702189	0.318440586	0.713923872
0.476275951	0.511683524	0.723584473
0.421607941	0.523271203	0.722056687
0.401269883	0.429340869	0.708671153
0.456162840	0.417658627	0.709546566
0.493144482	0.458883822	0.717298567
0.384268880	0.482543677	0.714877486
0.581141829	0.426656574	0.600338817
0.633248627	0.514315963	0.463505626
0.634540260	0.340566367	0.459990591
0.318403363	0.518374681	0.451620936
0.377839863	0.427883834	0.301737279
0.541395068	0.270987391	0.614560306
0.425273657	0.503499389	0.588527977
0.673077524	0.533664346	0.466697544
0.482246757	0.248207211	0.510996103
0.607607424	0.365069807	0.327597082
0.489887357	0.298142463	0.317112297
0.522246420	0.354369372	0.315394700
0.674248278	0.320947647	0.463170379
0.344372362	0.492116570	0.574704528
0.363551438	0.371955425	0.606384456
0.281373531	0.542867243	0.452099085
0.356558114	0.428059191	0.262770206
0.352257550	0.287832409	0.711901903
0.471108407	0.412441492	0.591587305
0.478651911	0.264713585	0.448038310
0.465702087	0.589760303	0.462404698
0.417043298	0.340693206	0.587501287
0.450408012	0.540188849	0.302291602
0.457365781	0.606261194	0.400191337
0.347277015	0.425905645	0.405480742
0.565557897	0.467856646	0.297853351
0.414930075	0.309020907	0.684373975
0.400376737	0.319387317	0.754576206
0.505445838	0.543677449	0.729038417
0.407735318	0.564438701	0.726676404
0.341563642	0.491774172	0.714087129
0.470402807	0.376929373	0.703193128
0.535782337	0.449390680	0.717944682
0.526651859	0.512712836	0.325803041
0.568291545	0.248591200	0.587846041
0.563767493	0.302105218	0.636016726
0.542584240	0.316568971	0.406686991
0.413868845	0.254337132	0.396834731
0.456531465	0.560895205	0.581176996
0.538083494	0.600402772	0.508619130
0.608678401	0.426379204	0.635167897
0.538364351	0.542309165	0.414087445
0.520794690	0.244042233	0.642568350
0.282356203	0.308354169	0.449121892
0.504833698	0.580568790	0.512298942
0.471297085	0.421407759	0.553411305
0.321566343	0.369588852	0.484311283
0.389884025	0.473313808	0.321001172
0.383560091	0.426177531	0.419297248
0.440619588	0.587341428	0.429931939
0.350995183	0.528143942	0.413810909
0.478120267	0.429329783	0.380875647
0.462050647	0.522822559	0.587869585
0.374954909	0.468472391	0.579453111
0.439704388	0.484548002	0.480755538
0.323050022	0.483825713	0.488492936
0.447838724	0.273296535	0.394490063
0.485184789	0.336818427	0.314900666
0.576192141	0.387976348	0.326999336
0.523144901	0.347816586	0.419214159
0.629243016	0.374877632	0.423063546
0.569950402	0.381600201	0.580155849
0.599565446	0.325359732	0.493691057
0.501747072	0.266224235	0.482445717
0.562040091	0.491751850	0.328605413
0.470887989	0.542463064	0.335356265
0.520395875	0.509746194	0.425361097
0.626950562	0.483181983	0.423740625
0.564527452	0.472324401	0.585602164
0.544267416	0.426464766	0.480585456
0.599488795	0.526139796	0.499374539
0.353936672	0.321451604	0.413792163
0.386941016	0.382124901	0.321921766
0.502403080	0.295168370	0.580324888
0.467322916	0.328602821	0.613794804
0.441235006	0.363443822	0.476723939
0.380661547	0.358193249	0.573423445
0.350593328	0.380970806	0.698889315
0.435683787	0.357590109	0.392971486
0.433785081	0.498532116	0.398062736
0.389760375	0.423027694	0.510336101
0.517013252	0.355613291	0.510151982
0.513659120	0.496106505	0.517311394
0.559866905	0.430789679	0.398763418

4

```
1.0
    25.0000000000     0.0000000000     0.0000000000
     0.0000000000    25.0000000000     0.0000000000
     0.0000000000     0.0000000000    25.0000000000
   C    H    O    S   Zr
  14   38   33    1    6
Direct
     0.330300003     0.314980000     0.434610009
     0.403120011     0.359310001     0.753820002
     0.522660017     0.524900019     0.741119981
     0.467779994     0.535539985     0.740769982
     0.451510012     0.445659995     0.710650027
     0.506200016     0.434659988     0.710139990
     0.541840017     0.474720001     0.725549996
     0.431750000     0.495840013     0.725070000
     0.588880002     0.410470009     0.591059983
     0.630739987     0.516939998     0.465299994
     0.644079983     0.346170008     0.439520001
     0.315690011     0.499269992     0.457850009
     0.377689987     0.433299989     0.297190011
     0.522300005     0.268999994     0.602270007
     0.425540000     0.478260010     0.588169992
     0.669030011     0.538869977     0.470380008
     0.504239976     0.237039998     0.479400009
     0.612190008     0.385630012     0.311789989
     0.498360008     0.313459992     0.292340010
     0.527540028     0.371100008     0.298729986
     0.685419977     0.329869986     0.439319998
     0.346379995     0.460649997     0.573520005
     0.353119999     0.316980004     0.561630011
     0.276870012     0.520439982     0.461970001
     0.355659992     0.436879992     0.258810014
     0.376720011     0.324640006     0.748340011
     0.473839998     0.392340004     0.584680021
     0.493739992     0.258049995     0.418430001
     0.458680004     0.579230011     0.476060003
     0.402150005     0.338290006     0.597389996
     0.442310005     0.550209999     0.311030000
     0.447450012     0.602909982     0.416610003
     0.349819988     0.416449994     0.399859995
     0.563229978     0.485040009     0.297450006
     0.443859994     0.346480012     0.763999999
     0.386970013     0.386200011     0.784609973
     0.550750017     0.556129992     0.753059983
     0.452980012     0.575049996     0.751969993
     0.388839990     0.503910005     0.723269999
     0.520210028     0.395489991     0.696850002
     0.584779978     0.466930002     0.725059986
     0.522329986     0.524820030     0.329380006
     0.526719987     0.225020006     0.604139984
     0.559000015     0.287290007     0.618830025
     0.552269995     0.322569996     0.382840008
     0.425980002     0.253459990     0.365099996
     0.455960006     0.536939979     0.586099982
     0.531130016     0.589190006     0.521929979
     0.617810011     0.409099996     0.624849975
     0.533330023     0.543739974     0.420819998
     0.488339990     0.280660003     0.627969980
     0.295509994     0.287369996     0.433129996
     0.499350011     0.566839993     0.523620009
     0.476119995     0.399639994     0.546270013
     0.332210004     0.345809996     0.475250006
     0.387419999     0.476819992     0.321590006
     0.386170000     0.417230010     0.413439989
     0.432669997     0.579450011     0.444099993
     0.346560001     0.516430020     0.421350002
     0.479530007     0.432770014     0.374089986
     0.461679995     0.498580009     0.591560006
     0.379049987     0.439770013     0.577419996
     0.439850003     0.471789986     0.481139988
     0.323709995     0.460619986     0.489870012
     0.458669990     0.274650007     0.364969999
     0.491470009     0.351709992     0.295819998
     0.579209983     0.406060010     0.314399987
     0.530950010     0.349949986     0.400319993
     0.635020018     0.384270012     0.407130003
     0.581680000     0.367590010     0.565750003
     0.611670017     0.324099988     0.471619993
     0.519529998     0.257510006     0.449860007
     0.558910012     0.506749988     0.329759985
     0.463129997     0.550000012     0.343959987
     0.517440021     0.509109974     0.428420007
     0.625739992     0.490819991     0.422019988
     0.566659987     0.455749989     0.583190024
     0.547619998     0.421359986     0.473010004
     0.597140014     0.521399975     0.503040016
     0.362769991     0.312370002     0.395929992
     0.389719993     0.386110008     0.311560005
     0.513970017     0.285430014     0.549260020
     0.434379995     0.357419997     0.653219998
     0.449570000     0.352750003     0.460779995
     0.389910012     0.329549998     0.560140014
     0.404610008     0.395040005     0.690680027
     0.443210006     0.356640011     0.377750009
     0.431459993     0.495389998     0.400469989
     0.396990001     0.403759986     0.501869977
     0.527010024     0.341820002     0.494610012
     0.514649987     0.482419997     0.517600000
     0.561380029     0.436529994     0.392580003
```

5

```
1.0
    25.0000000000     0.0000000000     0.0000000000
     0.0000000000    25.0000000000     0.0000000000
     0.0000000000     0.0000000000    25.0000000000
   C    O    H   Zr    S
  13   33   36    6    1
Direct
     0.319000006     0.331795007     0.445450008
     0.375149995     0.426663995     0.295906007
     0.316062003     0.517817020     0.446218997
     0.633795023     0.343434006     0.454142988
     0.630513012     0.516485989     0.456524014
     0.578410983     0.426200986     0.595115006
     0.435588986     0.479663014     0.811491013
     0.433638990     0.431104988     0.784094989
     0.413053989     0.430283010     0.732035995
     0.395480990     0.476978987     0.706857979
     0.397798002     0.525390029     0.734705985
     0.417638987     0.526690006     0.786857009
     0.368492991     0.329638004     0.732900023
     0.478495002     0.312016010     0.568117976
     0.466345012     0.345429987     0.691264987
     0.384629995     0.381152004     0.316412985
     0.352945000     0.320533007     0.409334004
     0.447396994     0.273351014     0.389118999
     0.383563012     0.355684996     0.566694021
     0.440250993     0.365202010     0.470021009
     0.321431011     0.368932992     0.480017990
     0.387178987     0.472404003     0.314503014
     0.381363004     0.426021993     0.413112015
     0.436903000     0.587343991     0.422984004
     0.347804010     0.527257025     0.407721996
     0.475937992     0.429661989     0.374435991
     0.459978014     0.524860024     0.582493007
     0.372824997     0.466540992     0.574994028
     0.437252015     0.485722005     0.474454999
     0.321705997     0.483850002     0.483608007
     0.469361007     0.423444003     0.547555983
     0.483743012     0.336825013     0.308780015
     0.574140012     0.388785005     0.320740998
     0.521830022     0.348217010     0.412838012
     0.627637029     0.376919001     0.416496009
     0.566172004     0.380890012     0.576089978
     0.599352002     0.328171015     0.488189012
     0.503241003     0.265837997     0.478251994
     0.559629023     0.492148012     0.321805000
     0.467388988     0.542499006     0.328393012
     0.517696023     0.510533988     0.418585002
     0.624521017     0.485033989     0.417050987
     0.562633991     0.471947014     0.579222977
     0.542217016     0.427763999     0.474599004
     0.500961006     0.581261992     0.505540013
     0.596717000     0.527917027     0.492590994
     0.489481002     0.316641986     0.605365992
     0.282640010     0.306345999     0.446808010
     0.413832992     0.253684014     0.391292006
     0.419248998     0.334791005     0.568069994
     0.379431009     0.371674001     0.602307975
     0.479568005     0.263534993     0.444882005
     0.469238013     0.421292990     0.586391985
     0.353406012     0.426348001     0.257194012
     0.278766990     0.541898012     0.447066009
     0.446916997     0.539737999     0.295356005
     0.341134995     0.488476992     0.569010973
     0.423426002     0.506295025     0.581303000
     0.673996985     0.324876994     0.457506001
     0.520669997     0.354773015     0.309356987
     0.488873005     0.298240989     0.311728001
     0.605870008     0.366311014     0.321319997
     0.483429998     0.252539009     0.509280980
     0.669957995     0.536670983     0.459403992
     0.461986989     0.589842975     0.455484986
     0.453514993     0.606392980     0.393249005
     0.524191976     0.512561977     0.318711996
     0.562955022     0.467144012     0.291889012
     0.535506010     0.543036997     0.406904012
     0.605861008     0.426156998     0.629963994
     0.533517003     0.602285981     0.502313972
     0.456012011     0.562294006     0.571590006
     0.345043004     0.425455004     0.399426997
     0.541314006     0.317382008     0.399435014
     0.451409012     0.481061995     0.852171004
     0.448635012     0.394524008     0.802690983
     0.381092012     0.475201994     0.665551007
     0.384644002     0.562165976     0.715179026
     0.419660002     0.564598978     0.808450997
     0.328608990     0.348134995     0.733258009
     0.384566993     0.326086015     0.773611009
     0.366815001     0.290304989     0.713418007
     0.434554011     0.357210994     0.387102008
     0.431273013     0.498392999     0.391265005
     0.389099985     0.423469007     0.503322005
     0.515820026     0.354523987     0.505205989
     0.557619989     0.431335986     0.392962009
     0.512242019     0.496338010     0.510363996
     0.411895007     0.370644003     0.691679001
```

6

```
1.0
     25.0000000000      0.0000000000      0.0000000000
      0.0000000000     25.0000000000      0.0000000000
      0.0000000000      0.0000000000     25.0000000000
   C    O   Zr    H    S
  14   34    6   38    1
Direct
     0.318818003         0.332635999         0.443699002
     0.375624001         0.426703006         0.295681000
     0.316289008         0.518016994         0.445598006
     0.634974003         0.344514012         0.452713013
     0.630729973         0.517499983         0.455648988
     0.581194997         0.428066999         0.593216002
     0.514895976         0.245444998         0.617509007
     0.444546998         0.473663002         0.822381020
     0.439323992         0.425633997         0.794494987
     0.406762004         0.423965007         0.749005020
     0.381024987         0.469617993         0.729888022
     0.386866003         0.517593026         0.758078992
     0.418172985         0.519595981         0.804305017
     0.367924005         0.323215008         0.763045013
     0.478733987         0.319103003         0.574223995
     0.475336999         0.260545999         0.579324007
     0.385363996         0.381224990         0.316114992
     0.353767008         0.320751995         0.408643007
     0.449021995         0.273552001         0.391896009
     0.381518990         0.357219994         0.565518022
     0.441109985         0.366174012         0.469733000
     0.320657015         0.369856000         0.478105009
     0.387591004         0.472498000         0.314184994
     0.381924987         0.426331013         0.412817001
     0.437009990         0.587733984         0.422710001
     0.348232001         0.527562976         0.407240987
     0.476419002         0.429910004         0.373946995
     0.460496992         0.523962975         0.582337976
     0.374464005         0.468629986         0.573991001
     0.437747002         0.485590011         0.474337995
     0.321758002         0.484043002         0.482942998
     0.470124990         0.422183990         0.547861993
     0.484194010         0.335759997         0.309287995
     0.573843002         0.389308989         0.319815993
     0.522862971         0.349056989         0.412726015
     0.628473997         0.378118992         0.415226012
     0.569949985         0.382598996         0.574136019
     0.600888014         0.329239011         0.487109989
     0.505846977         0.268642008         0.480141014
     0.559518993         0.492749989         0.320859998
     0.467474014         0.542819023         0.328004003
     0.517804980         0.511201978         0.417962998
     0.624692023         0.486220002         0.416004002
     0.563394010         0.473426998         0.578441024
     0.542648971         0.428811997         0.473841995
     0.501403987         0.581834972         0.505253017
     0.597027004         0.528818011         0.491786987
     0.356985003         0.372729003         0.669932008
     0.435086012         0.357434988         0.387279004
     0.431549996         0.498524994         0.391137987
     0.388651997         0.423664004         0.503675997
     0.516951025         0.355987996         0.504139006
     0.557982981         0.432307005         0.392098993
     0.512104988         0.497245014         0.510176003
     0.282142013         0.307615995         0.443921000
     0.415560991         0.253814012         0.394854993
     0.417048007         0.337895989         0.569516003
     0.370296001         0.366683990         0.602617025
     0.481566995         0.265460014         0.447133988
     0.468932986         0.420507014         0.586730003
     0.353653997         0.426322997         0.257081985
     0.279004991         0.542122006         0.446247995
     0.446803004         0.539978981         0.295096993
     0.342738986         0.490821004         0.569673002
     0.423370987         0.505016029         0.581587970
     0.675161004         0.325812012         0.455509990
     0.520810008         0.354454011         0.309385985
     0.490123004         0.297374010         0.313178003
     0.605822027         0.367190003         0.320230007
     0.489071995         0.251161009         0.511623025
     0.670240998         0.537585974         0.458552003
     0.462103009         0.590395987         0.455148011
     0.453604013         0.606649995         0.392874986
     0.523968995         0.513177991         0.318093002
     0.562415004         0.467539012         0.291065007
     0.535356998         0.543767989         0.406071007
     0.609780014         0.428759009         0.627216995
     0.534171999         0.602454007         0.501582980
     0.455776989         0.561833024         0.573782027
     0.345676988         0.425783008         0.398948997
     0.542243004         0.317912996         0.399897009
     0.555478990         0.255602986         0.603527009
     0.507049024         0.265157014         0.656135023
     0.510639012         0.201781005         0.621523976
     0.469406009         0.475214988         0.858245015
     0.460505992         0.389988005         0.808306992
     0.357169986         0.467058003         0.693367004
     0.366905004         0.553629994         0.743646026
     0.422430992         0.557107985         0.826272011
     0.329892009         0.342528999         0.773503006
     0.394537002         0.320553988         0.797873974
     0.361146986         0.283322990         0.745971024
     0.399845988         0.362773001         0.711055994
```

TS2

1.0

25.0000000000	0.0000000000	0.0000000000
0.0000000000	25.0000000000	0.0000000000
0.0000000000	0.0000000000	25.0000000000

C	H	O	S	Zr
14	38	34	1	6

Direct

0.320428014	0.330177993	0.445944995
0.505270004	0.474346012	0.717565000
0.369657010	0.351846993	0.776341021
0.474938989	0.520413995	0.726085007
0.424216002	0.422345012	0.715095997
0.479911000	0.424836993	0.711776018
0.635416985	0.338871002	0.457033992
0.419055998	0.517513990	0.727985024
0.582665026	0.420437008	0.599898994
0.633691013	0.512596011	0.465925992
0.318482012	0.514880002	0.455722004
0.378351986	0.430566013	0.302096009
0.542382002	0.264438003	0.610239983
0.393348008	0.468398005	0.722168028
0.345344990	0.484899014	0.579095006
0.425805002	0.497016996	0.590438008
0.482836008	0.244764999	0.505244017
0.608578026	0.367808998	0.326047987
0.491304994	0.300972998	0.313118011
0.523214996	0.357401013	0.313089997
0.675108016	0.319085002	0.459383994
0.673487008	0.531965971	0.469671994
0.450064003	0.543021977	0.306430012
0.357098013	0.431959987	0.263145000
0.348257989	0.313625991	0.779402018
0.479786009	0.262924999	0.442748010
0.465728015	0.587339997	0.467599005
0.417331994	0.334149987	0.584316015
0.347932994	0.424778014	0.405800998
0.361292005	0.359813988	0.604220986
0.281233996	0.538986981	0.457143992
0.457379997	0.605048001	0.405712008
0.472526014	0.403099000	0.590113997
0.565651000	0.471673995	0.299082994
0.457219988	0.554871023	0.584281981
0.526510000	0.515200973	0.328722000
0.494839996	0.559078991	0.730130970
0.395484000	0.553819001	0.733824015
0.349745005	0.465505004	0.722217023
0.502783000	0.388453990	0.703804016
0.548717022	0.477120012	0.714362025
0.568220973	0.240750000	0.583550990
0.342920005	0.385448009	0.785669982
0.543522000	0.316370010	0.403124988
0.415531009	0.254063010	0.390772998
0.538396001	0.596670985	0.513705015
0.610767007	0.419265985	0.634302974
0.538406014	0.541673005	0.417874008
0.404769987	0.352016002	0.802774012
0.283683985	0.305290997	0.444822013
0.566164017	0.294788986	0.631341994
0.520744026	0.238578007	0.638517022
0.505150020	0.576767981	0.517064989
0.472209007	0.416209996	0.553065002
0.322389990	0.365565985	0.482154012
0.630093992	0.374404997	0.421270996
0.384207010	0.424728990	0.419636995
0.440467000	0.585893989	0.435229987
0.350937009	0.526371002	0.418289989
0.323453009	0.479057014	0.491313010
0.462797999	0.516776979	0.590912998
0.377166986	0.462588012	0.581251025
0.449440002	0.273203999	0.389180988
0.486281991	0.339657009	0.311942995
0.577167988	0.390736997	0.325816989
0.524115980	0.347216010	0.416640997
0.478675991	0.429751992	0.381098002
0.390011013	0.475400001	0.322948009
0.440138012	0.481265008	0.482917011
0.469455004	0.323673010	0.609678984
0.600013018	0.522984982	0.502299011
0.502655029	0.263424009	0.477333993
0.562052011	0.494399011	0.330691010
0.470607013	0.544386029	0.339507997
0.571497023	0.375983000	0.578568995
0.627371013	0.482894003	0.425067008
0.565473974	0.466439009	0.586930990
0.545224011	0.423817992	0.480610996
0.520563006	0.508728027	0.428180009
0.355352998	0.319943994	0.410272986
0.387775987	0.384193987	0.320621997
0.442133993	0.360439003	0.474691987
0.381121010	0.351410002	0.570725977
0.600553989	0.322560996	0.490319997
0.342610002	0.365168989	0.674517989
0.504199982	0.289947987	0.576120973
0.392603010	0.357814014	0.707633018
0.517850995	0.351897001	0.507872999
0.514093995	0.491991997	0.519537985
0.436661005	0.357437015	0.390875012
0.433894992	0.497961998	0.400929004
0.390309989	0.418316990	0.510828018
0.560406983	0.430923998	0.398939013

7

1.0

25.0000000000	0.0000000000	0.0000000000
0.0000000000	25.0000000000	0.0000000000
0.0000000000	0.0000000000	25.0000000000

C	O	Zr	H	S
14	34	6	38	1

Direct

0.317746013	0.331162989	0.440405011
0.372424006	0.438932002	0.299515992
0.317580014	0.516676009	0.458456010
0.632683992	0.337258995	0.443482995
0.632201016	0.509831011	0.462561011
0.582624018	0.409474999	0.592351973
0.497485012	0.286514997	0.612348974
0.438104004	0.505680978	0.803035021
0.440142006	0.451499999	0.789942026
0.412137002	0.433571994	0.744929016
0.383094996	0.468439996	0.712320983
0.381563008	0.522590995	0.725965977
0.408605009	0.541038990	0.771263003
0.361811012	0.336811006	0.771283984
0.483680010	0.297289997	0.558241010
0.463858992	0.341571987	0.746427000
0.381713986	0.391599000	0.315425992
0.350755990	0.323136002	0.402628005
0.443980008	0.276762009	0.376112014
0.385955006	0.342642009	0.563392997
0.440014005	0.360179007	0.466001987
0.321686000	0.364262015	0.478751004
0.385349989	0.482576996	0.322131008
0.380939990	0.427102000	0.416085988
0.439296007	0.586320996	0.439736009
0.348946005	0.529255986	0.420547009
0.474752009	0.433306009	0.375930011
0.463284999	0.508231997	0.592029989
0.375092000	0.453112990	0.580078006
0.438621014	0.479956001	0.481380999
0.322881013	0.478911012	0.492036015
0.471464992	0.410571009	0.548025012
0.479815990	0.347445011	0.301371008
0.571382999	0.396315008	0.316554993
0.520144999	0.348084986	0.405386001
0.626505971	0.374624997	0.409676999
0.570517004	0.366356999	0.568827987
0.598744988	0.319409996	0.476767004
0.501998007	0.259797007	0.462435007
0.558327973	0.499300003	0.327690005
0.467103988	0.550007999	0.340772003
0.518514991	0.509087026	0.426609993
0.624970019	0.482463986	0.420329005
0.565428019	0.456344992	0.582286000
0.542927980	0.421243995	0.474061996
0.504471004	0.571566999	0.520281971
0.599252999	0.518352985	0.500164986
0.395599991	0.357365012	0.674443007
0.432655007	0.360514998	0.382647008
0.431421012	0.500648022	0.399991989
0.390228987	0.415789008	0.505558014
0.516223013	0.345768005	0.498483986
0.556828976	0.432103008	0.392841995
0.514230013	0.486526996	0.516708016
0.280797988	0.306540996	0.439702988
0.410106003	0.257573992	0.377472013
0.420399010	0.321390986	0.560895979
0.382714987	0.352804005	0.601215005
0.477393001	0.261137992	0.429735988
0.470705003	0.405636013	0.586623013
0.349940002	0.442523986	0.261391014
0.281004995	0.541548014	0.462435007
0.445906997	0.550689995	0.308082998
0.344628990	0.476880997	0.574678004
0.426369995	0.490206987	0.590120971
0.672429979	0.317434013	0.443742007
0.517096996	0.364591002	0.303045005
0.484263986	0.308710992	0.299811989
0.602437973	0.372969002	0.314832002
0.482748985	0.243709996	0.492460996
0.672104001	0.528910995	0.466630012
0.464924008	0.585359991	0.471852988
0.456005991	0.607340991	0.411417007
0.523144007	0.520359993	0.327279001
0.560772002	0.477302998	0.295428991
0.536642015	0.542293012	0.417744011
0.611377001	0.406269014	0.626016974
0.537441015	0.592114985	0.518494010
0.459408998	0.546651006	0.585600019
0.344422996	0.428207994	0.402967989
0.538730979	0.318253011	0.388821006
0.539628029	0.274031997	0.615691006
0.492029011	0.321927994	0.638145983
0.471740991	0.254346013	0.628169000
0.459742993	0.520385981	0.837975025
0.463743001	0.423415005	0.813628018
0.363810003	0.453512996	0.676056027
0.359216005	0.550467014	0.700847983
0.407058001	0.583398998	0.781708002
0.323664993	0.356193990	0.761533022
0.372936994	0.343497008	0.813197017
0.360289007	0.293828011	0.762247026
0.413177013	0.363997012	0.729514003

8

```
1.0
    25.0000000000     0.0000000000     0.0000000000
     0.0000000000    25.0000000000     0.0000000000
     0.0000000000     0.0000000000    25.0000000000
   C   H   O   S  Zr
  13  36  34   1   6
Direct
    0.324077010    0.330332011    0.447012991
    0.371802002    0.336923987    0.775551021
    0.478195995    0.511940002    0.725543022
    0.427506000    0.522055984    0.746622026
    0.400974005    0.432413012    0.723981023
    0.451595008    0.421826005    0.702551007
    0.490265995    0.461923003    0.703598976
    0.388415009    0.482111990    0.746077001
    0.585433006    0.423781008    0.597234011
    0.634590983    0.516655982    0.461059988
    0.638782024    0.343243003    0.455146015
    0.319914013    0.515626013    0.452524006
    0.379099995    0.428671986    0.299475014
    0.505196989    0.312718004    0.604469001
    0.427935988    0.501420021    0.586995006
    0.674015999    0.536844015    0.464246988
    0.488943011    0.250135005    0.508584023
    0.610800982    0.369345009    0.323403001
    0.493914992    0.301216006    0.311385006
    0.525452971    0.357870996    0.310784996
    0.678852022    0.324279994    0.457895994
    0.346673995    0.482924998    0.578776002
    0.375299007    0.356952995    0.604083002
    0.282406986    0.539341986    0.454192996
    0.357275009    0.429078013    0.260807008
    0.342133999    0.304533988    0.776210010
    0.471547008    0.418989003    0.589339972
    0.484183997    0.263141006    0.444546998
    0.465627998    0.589242995    0.461623996
    0.424890995    0.331131995    0.571927011
    0.450266004    0.542003989    0.300980002
    0.457305998    0.606231987    0.399464995
    0.349474013    0.424787015    0.403214008
    0.567167997    0.471071005    0.295727015
    0.411585987    0.321157992    0.765958011
    0.372018993    0.358671993    0.813641012
    0.508542001    0.543317020    0.725522995
    0.418139994    0.561263025    0.763391972
    0.348334014    0.489116013    0.761847973
    0.460056007    0.382849991    0.684706986
    0.529646993    0.454863012    0.686335981
    0.527592003    0.515083015    0.323774993
    0.546415985    0.318140000    0.400005996
    0.419081002    0.254103988    0.390316993
    0.458887994    0.558813989    0.577548981
    0.537468016    0.600790977    0.508324027
    0.614997029    0.423698008    0.630472004
    0.539219022    0.543649018    0.412542999
    0.287838995    0.304708004    0.447643995
    0.504911005    0.579786003    0.511637986
    0.474601001    0.420736998    0.550616026
    0.326169997    0.366739005    0.482336015
    0.390652001    0.474063009    0.319207996
    0.385868996    0.425386995    0.416720003
    0.440571010    0.587144017    0.429122001
    0.351469994    0.526504993    0.414180994
    0.480298996    0.430707991    0.377741992
    0.464334995    0.521245003    0.587100983
    0.379583001    0.462119997    0.579828024
    0.441399992    0.484162986    0.478964001
    0.325978994    0.480666995    0.488867998
    0.452598006    0.273929000    0.388983011
    0.488608003    0.339848995    0.309819013
    0.579060018    0.391815990    0.323027015
    0.526874006    0.348639011    0.414077014
    0.632579029    0.377584010    0.418336004
    0.572481990    0.378412008    0.578846991
    0.604457974    0.327423006    0.489149004
    0.508005977    0.265017003    0.477872014
    0.563373029    0.495104015    0.326359004
    0.470732003    0.544152975    0.334055990
    0.521506011    0.510833979    0.423480004
    0.628602982    0.485946000    0.420985997
    0.568418026    0.469521999    0.582925975
    0.547156990    0.427161992    0.477759004
    0.600826025    0.527365029    0.497361004
    0.358238995    0.319999993    0.410766989
    0.389162004    0.382789999    0.318836987
    0.487551987    0.308795989    0.570084989
    0.356103987    0.351182997    0.673664987
    0.445499986    0.363914996    0.472081006
    0.389660001    0.350769013    0.567888975
    0.300283015    0.404689997    0.738040984
    0.351152003    0.381323993    0.723322988
    0.439289987    0.357780010    0.388998985
    0.434861988    0.498661995    0.396250010
    0.393534988    0.420884013    0.507528007
    0.520922005    0.353363007    0.506213009
    0.516094029    0.494924009    0.514689982
    0.561936021    0.432579994    0.395980000
```

9

1.0

25.0000000000	0.0000000000	0.0000000000
0.0000000000	25.0000000000	0.0000000000
0.0000000000	0.0000000000	25.0000000000

C O Zr H P

13 35 6 35 1

Direct

0.312475115	0.329460025	0.438830048
0.377136260	0.427763611	0.295076132
0.313007921	0.516676724	0.442773521
0.626933753	0.336882919	0.464594096
0.627811611	0.510287285	0.466786623
0.569015682	0.424595177	0.602142990
0.525343060	0.275105774	0.631887972
0.325474471	0.235301957	0.720878959
0.358582616	0.239540577	0.676067472
0.354394346	0.283595830	0.642003834
0.316854388	0.323820889	0.653018594
0.284090757	0.319953531	0.698434949
0.288351029	0.275626838	0.732078910
0.462537229	0.315101445	0.573887408
0.508375406	0.277716041	0.577377379
0.385509551	0.381828099	0.315072894
0.347978890	0.320266843	0.403987199
0.441713691	0.272132277	0.387956262
0.432433814	0.363366961	0.471421182
0.313011467	0.365751714	0.474708796
0.389024675	0.473019779	0.314958066
0.378563821	0.425172806	0.412327737
0.436769962	0.585883319	0.424656361
0.346637130	0.526907563	0.406326592
0.474574357	0.427952260	0.378034502
0.458036423	0.528484583	0.586659849
0.360972971	0.488888681	0.575222492
0.432169944	0.485600710	0.477323681
0.317009866	0.481973886	0.479982972
0.458502591	0.421869993	0.551544905
0.484004617	0.336240053	0.311640114
0.574765861	0.384693444	0.329126894
0.516645968	0.345378041	0.418186128
0.622825265	0.370916188	0.427152365
0.557237327	0.379392266	0.582749486
0.591253042	0.322200239	0.497556627
0.494251460	0.263302445	0.477290303
0.562515616	0.488101840	0.329994708
0.470509708	0.540539443	0.332056075
0.516235769	0.507178247	0.424316347
0.622672915	0.478493035	0.427505553
0.553800583	0.470256209	0.585882664
0.534700751	0.423611522	0.481544793
0.498986632	0.579509974	0.508116603
0.592686057	0.522919178	0.501128018
0.304036498	0.431904733	0.637447953
0.258894205	0.366521001	0.571627736
0.359180868	0.378770828	0.569849014
0.431645662	0.356245399	0.387546420
0.430907011	0.497391313	0.393022656
0.384079397	0.421809286	0.501980007
0.507522464	0.349707007	0.512375176
0.555443704	0.426894456	0.400870681
0.506747663	0.493730307	0.515351832
0.276643485	0.303254068	0.438100576
0.407518417	0.253478169	0.387968540
0.337797761	0.469202250	0.604447544
0.470561594	0.261637598	0.443866074
0.452990890	0.414073795	0.589325964
0.356671840	0.428402692	0.255703449
0.275534838	0.540453553	0.442270130
0.451781303	0.538313985	0.297965288
0.335825324	0.511244953	0.555346370
0.419523150	0.516836166	0.586664021
0.666493535	0.317147166	0.468788564
0.521513164	0.352905840	0.314406991
0.487711400	0.297425538	0.313293934
0.605642855	0.361149818	0.331220359
0.480283231	0.239892840	0.505100131
0.667575657	0.529498339	0.471217394
0.461166292	0.588324964	0.457780004
0.454156429	0.604964614	0.395388186
0.527835011	0.509077311	0.324881852
0.567267358	0.463215709	0.300169021
0.535207629	0.539339423	0.413533628
0.595519662	0.424590111	0.637758017
0.532566369	0.598899901	0.505139649
0.459652066	0.565787554	0.574287355
0.342745930	0.425616801	0.397373885
0.535970628	0.313996017	0.405803829
0.536617339	0.314850658	0.646725476
0.493166566	0.257689506	0.656373858
0.560470581	0.248600781	0.631322682
0.255541146	0.351755530	0.707429588
0.262729347	0.272556663	0.767300785
0.328666240	0.200616643	0.747266769
0.387964636	0.208355159	0.667495608
0.381015360	0.287654281	0.607610524
0.270188928	0.363043308	0.533648968
0.310302019	0.379523724	0.608004570

10

1.0

25.0000000000	0.0000000000	0.0000000000
0.0000000000	25.0000000000	0.0000000000
0.0000000000	0.0000000000	25.0000000000

C	H	O	Zr	P
18	42	38	6	2

Direct

0.327568979	0.324641035	0.455359412
0.582442105	0.422636389	0.606215423
0.632896829	0.518072484	0.468579417
0.641391320	0.346377862	0.459183109
0.318644867	0.510119941	0.463629615
0.377116722	0.427083568	0.308827795
0.416837862	0.393135610	0.855459841
0.417494512	0.400081290	0.800141533
0.445109596	0.443589776	0.777755059
0.471842574	0.479919394	0.811146953
0.470885199	0.472682700	0.866505865
0.443468291	0.429355270	0.888664942
0.553738922	0.322715672	0.833062179
0.535592044	0.312730061	0.781268528
0.491551341	0.278848427	0.772927844
0.466172312	0.254948816	0.816888946
0.484832128	0.264750968	0.868621762
0.528481422	0.298660009	0.876754721
0.502310191	0.291651671	0.606564564
0.424270232	0.505324918	0.590406253
0.671879727	0.539006435	0.472065770
0.495217786	0.250188280	0.511769952
0.610707636	0.373124429	0.327116195
0.495159481	0.302674582	0.315637975
0.525510568	0.359962036	0.315914653
0.682083242	0.328780498	0.461247109
0.342241124	0.477947347	0.580442316
0.384400638	0.350295465	0.613541940
0.280906921	0.533301840	0.466557542
0.354285743	0.427669264	0.270767603
0.475707611	0.412440931	0.598255120
0.491131642	0.261279612	0.447833552
0.462163885	0.587147534	0.471676521
0.431762996	0.325929706	0.577045540
0.445515415	0.542205669	0.310922041
0.451863927	0.604839301	0.410097409
0.349616388	0.420555757	0.413296728
0.563981351	0.474140792	0.302512184
0.524079801	0.516827257	0.332044844
0.548798401	0.319311021	0.404622164
0.423594742	0.251863381	0.395138665
0.461379727	0.559044890	0.577160363
0.532900468	0.600533863	0.521478202
0.606994205	0.422141230	0.643100449
0.536272778	0.544115868	0.421515537
0.291929290	0.298203867	0.456308344
0.395744676	0.359064682	0.872736560
0.396847295	0.371548691	0.774339140
0.492965464	0.513807449	0.793612035
0.491462378	0.501174018	0.892436000
0.442880170	0.423700602	0.931992108
0.379512700	0.456276661	0.647558497
0.476662016	0.517057482	0.659390703
0.587793683	0.349303139	0.839379478
0.555465720	0.330952029	0.746894387
0.431964763	0.228581827	0.810481630
0.465092546	0.245866832	0.902709305
0.542897014	0.306397947	0.917242628
0.444010164	0.187833338	0.691737444
0.448095032	0.353184544	0.685857719
0.501647671	0.577409338	0.521503114
0.475435109	0.418177973	0.559132917
0.329825825	0.360264010	0.491554410
0.388136636	0.472373366	0.329106472
0.386336291	0.421801940	0.425839994
0.436488825	0.584743448	0.439826770
0.349142423	0.522015706	0.424845238
0.479666130	0.430385945	0.385301225
0.460346548	0.523494290	0.595062470
0.375400789	0.459178207	0.589213287
0.440513262	0.481368812	0.488610020
0.326031943	0.474560468	0.499249484
0.456199733	0.273047953	0.392717019
0.489065201	0.341228154	0.315112556
0.578530484	0.394938929	0.328077462
0.528753301	0.349150486	0.419394522
0.633626178	0.380928486	0.422804932
0.574614562	0.378277806	0.583455771
0.608012568	0.329102963	0.493327216
0.515416436	0.262668976	0.480501060
0.560177568	0.497433751	0.333723022
0.466430794	0.544268114	0.343726515
0.519518271	0.510626488	0.431901070
0.626978530	0.488760648	0.427506865
0.565192511	0.468562028	0.591683529
0.547669518	0.426681930	0.483958475
0.599368884	0.526617060	0.505725121
0.360989368	0.316023180	0.418066225
0.388751514	0.381140680	0.327100801
0.488721665	0.305153907	0.571559255
0.447907815	0.361028954	0.478959756
0.394822705	0.345689475	0.576290078
0.471626352	0.405697395	0.675998541
0.477631476	0.505649933	0.698044234
0.385519318	0.458509022	0.689566185
0.513929968	0.261505198	0.667180827
0.428737743	0.316143955	0.691970423
0.427570932	0.219375144	0.707680991
0.440927900	0.355973465	0.395501444
0.433034371	0.496651132	0.405517756
0.396328364	0.415284068	0.515760793
0.524305237	0.350954499	0.512855471
0.516564721	0.491436504	0.522367475
0.561656770	0.433696812	0.402058518
0.445593481	0.451716403	0.706530048
0.468557684	0.269146909	0.705230599

11

```
1.0
    25.0000000000         0.0000000000         0.0000000000
     0.0000000000        25.0000000000         0.0000000000
     0.0000000000         0.0000000000        25.0000000000
   C    O   Zr    H
   7   33    6   30
Direct
     0.324268013         0.327641010         0.450246006
     0.375546992         0.420744002         0.296817005
     0.314808995         0.513904989         0.445178002
     0.639946997         0.354609013         0.455186009
     0.629127979         0.522508979         0.453440994
     0.578921974         0.431603014         0.597259998
     0.524689972         0.244590998         0.624804974
     0.481391996         0.326819986         0.611222029
     0.386433989         0.376125991         0.318365991
     0.357580006         0.317007989         0.413583010
     0.453198999         0.273842007         0.388420999
     0.379743993         0.363918990         0.578803003
     0.443213999         0.366878003         0.472409010
     0.326238990         0.365640014         0.484118998
     0.386422992         0.467354000         0.314159006
     0.382432014         0.423536986         0.413713992
     0.433028996         0.586654007         0.419910014
     0.345871001         0.523528993         0.406197011
     0.477010012         0.429367006         0.374422997
     0.461162001         0.526244998         0.580731988
     0.375283003         0.468425006         0.575668991
     0.437155008         0.486418992         0.473446995
     0.322084993         0.481142998         0.483415991
     0.470528007         0.423729986         0.548847020
     0.487262011         0.337345004         0.309864998
     0.577004015         0.391404986         0.320033014
     0.525970995         0.350048989         0.414999008
     0.631108999         0.384229988         0.414909989
     0.565289021         0.384534001         0.582252026
     0.607218981         0.340745002         0.491376996
     0.510137022         0.280007005         0.501457989
     0.557524025         0.494078010         0.318913996
     0.463627994         0.540939987         0.325450987
     0.516161978         0.512857974         0.415843010
     0.623574972         0.491578013         0.413592994
     0.564612985         0.475641012         0.577280998
     0.543712020         0.432074010         0.472698003
     0.498957008         0.583777010         0.502277017
     0.595355988         0.532455981         0.490065008
     0.481267989         0.277269989         0.642965019
     0.437913001         0.354885012         0.389133990
     0.430106014         0.496589988         0.389310002
     0.390816003         0.423662007         0.503310978
     0.520456016         0.358658999         0.505228996
     0.558682024         0.434464991         0.390684009
     0.511838973         0.499671012         0.507404983
     0.288915008         0.300980985         0.453226000
     0.422104001         0.251201004         0.394329011
     0.412515014         0.346536994         0.592256010
     0.372233003         0.393824011         0.603266001
     0.493066996         0.262717992         0.470780998
     0.469460011         0.429352999         0.587305009
     0.353529990         0.418938011         0.258325011
     0.276618987         0.536512017         0.445764989
     0.442999005         0.536889970         0.292643011
     0.343017012         0.489966005         0.572045982
     0.424225986         0.507616997         0.581053019
     0.681180000         0.338486999         0.458992004
     0.523763001         0.355661988         0.309648991
     0.492606014         0.298781991         0.312990010
     0.608669996         0.368909001         0.322003990
     0.668015003         0.543712020         0.456375003
     0.458169997         0.590653002         0.451927006
     0.448357999         0.606160998         0.389800012
     0.521382987         0.513194025         0.315719992
     0.562062025         0.469422996         0.288902014
     0.532449007         0.545925021         0.403539985
     0.606178999         0.433537006         0.632251024
     0.530866981         0.605627000         0.498243988
     0.456611991         0.564028025         0.571493983
     0.346179008         0.421671003         0.399958998
     0.543079019         0.316935986         0.403863013
     0.519401014         0.207363993         0.647750020
     0.522059023         0.237189993         0.581529021
     0.563621998         0.262740999         0.635109007
     0.513078988         0.346446007         0.624656022
```

5 References

1. Liu, X.; Kirlikovali, K. O.; Chen, Z.; Ma, K.; Idrees, K. B.; Cao, R.; Zhang, X.; Islamoglu, T.; Liu, Y.; Farha, O. K., Small Molecules, Big Effects: Tuning Adsorption and Catalytic Properties of Metal–Organic Frameworks. *Chem. Mater.* **2021,** *33* (4), 1444-1454.
2. Howarth, A. J.; Peters, A. W.; Vermeulen, N. A.; Wang, T. C.; Hupp, J. T.; Farha, O. K., Best Practices for the Synthesis, Activation, and Characterization of Metal–Organic Frameworks. *Chem. Mater.* **2017,** *29* (1), 26-39.
3. Furukawa, H.; Gándara, F.; Zhang, Y.-B.; Jiang, J.; Queen, W. L.; Hudson, M. R.; Yaghi, O. M., Water Adsorption in Porous Metal–Organic Frameworks and Related Materials. *J. Am. Chem. Soc.* **2014,** *136* (11), 4369-4381.
4. Liao, Y.; Sheridan, T. R.; Liu, J.; Lu, Z.; Ma, K.; Yang, H.; Farha, O. K.; Hupp, J. T., Probing the Mechanism of Hydrolytic Degradation of Nerve Agent Simulant with Zirconium-Based Metal–Organic Frameworks. *ACS Catal.* **2024,** *14* (1), 437-448.
5. Kresse, G.; Furthmüller, J., Efficient iterative schemes for ab initio total-energy calculations using a plane-wave basis set. *Phys. Rev. B* **1996,** *54* (16), 11169-11186.
6. Perdew, J. P.; Burke, K.; Ernzerhof, M., Generalized Gradient Approximation Made Simple. *Phys. Rev. Lett.* **1996,** *77* (18), 3865-3868.
7. Kresse, G.; Joubert, D., From ultrasoft pseudopotentials to the projector augmented-wave method. *Phys. Rev. B* **1999,** *59* (3), 1758-1775.
8. Blöchl, P. E., Projector augmented-wave method. *Phys. Rev. B* **1994,** *50* (24), 17953-17979.
9. Ingram, Z. J.; Lander, C. W.; Oliver, M. C.; Altınçekiç, N. G.; Huang, L.; Shao, Y.; Noh, H., Hydrogen-Atom Binding Energy of Structurally Well-defined Cerium Oxide Nodes at the Metal−Organic Framework-Liquid Interfaces. *J. Phys. Chem. C* **2024,** *128* (23), 9556–9565.
10. Grimme, S.; Antony, J.; Ehrlich, S.; Krieg, H., A consistent and accurate ab initio parametrization of density functional dispersion correction (DFT-D) for the 94 elements H-Pu. *J. Chem. Phys.* **2010,** *132* (15), 154104.
11. Grimme, S.; Ehrlich, S.; Goerigk, L., Effect of the damping function in dispersion corrected density functional theory. *J. Comput. Chem.* **2011,** *32* (7), 1456-1465.
12. Henkelman, G.; Uberuaga, B. P.; Jónsson, H., A climbing image nudged elastic band method for finding saddle points and minimum energy paths. *J. Chem. Phys.* **2000,** *113* (22), 9901-9904.
13. Henkelman, G.; Jónsson, H., A dimer method for finding saddle points on high dimensional potential surfaces using only first derivatives. *J. Chem. Phys.* **1999,** *111* (15), 7010-7022.
14. Wang, V.; Xu, N.; Liu, J.-C.; Tang, G.; Geng, W.-T., VASPKIT: A user-friendly interface facilitating high-throughput computing and analysis using VASP code. *Comput. Phys. Commun.* **2021,** *267*, 108033.
15. Grimme, S., Supramolecular Binding Thermodynamics by Dispersion-Corrected Density Functional Theory. *Chem. Eur. J.* **2012,** *18* (32), 9955-9964.
16. Masood, Z.; Nguyen, Q. P.; Wang, B., Computational Design of Metal-Exchanged MFI Zeolites for Coupling CO_2–Ethylene to Form Acrylic Acid. *ACS Sustainable Chem. Eng.* **2024,** *12* (18), 6960-6968.

17. Mathew, K.; Sundararaman, R.; Letchworth-Weaver, K.; Arias, T. A.; Hennig, R. G., Implicit solvation model for density-functional study of nanocrystal surfaces and reaction pathways. *The Journal of Chemical Physics* **2014,** *140* (8), 084106.
18. Mathew, K.; Kolluru, V. S. C.; Mula, S.; Steinmann, S. N.; Hennig, R. G., Implicit self-consistent electrolyte model in plane-wave density-functional theory. *The Journal of Chemical Physics* **2019,** *151* (23), 234101.
